# Pulsed Electromagnetic Fields and Bioimpedance Monitoring of Peripheral Circulation: A Scoping Review and Proposed Adaptive Control Framework

**DOI:** 10.3390/bioengineering13101160

**Published:** 2026-10-03

**Authors:** Ahmad Hammoud, Andrey Briko, Vladislava Kapravchuk, Artem Malakhov, Sergey Shchukin

**Affiliations:** 1 Petrovsky National Research Center of Surgery, Moscow 119435, Russia; briko@bmstu.ru (A.B.); kapravchuk@bmstu.ru (V.K.); schookin@bmstu.ru (S.S.); 2 Department of Medical and Technical Information Technology, Bauman Moscow State Technical University, Moscow 105005, Russia; 3 LLC PrimeKey, Moscow 125047, Russia; a.malahov@primekey.tech

**Keywords:** pulsed electromagnetic fields, PEMF, peripheral circulation, bioimpedance, impedance plethysmography, rheovasography, hemodynamic monitoring, pulse wave analysis, adaptive control, scoping review

## Abstract

Evidence for pulsed electromagnetic fields (PEMFs) in peripheral circulatory dysfunction is heterogeneous, while limb bioimpedance may index segmental pulsatile hemodynamics. This scoping review mapped PEMF effects, bioimpedance-based vascular assessment, and adaptive stimulation architectures. Scopus, PubMed, and Web of Science were searched in July 2026, supplemented by IEEE Xplore during revision (PRISMA-ScR reporting; no registered protocol). The main search used AI-assisted sequential investigator review; IEEE records underwent independent duplicate human screening. The main search yielded 1750 unique records and 30 core reports; IEEE Xplore added two technical reports. The combined corpus comprised 32 reports, conservatively grouped into 30 study/report clusters. It contained 15 direct clinical, six clinical support/proxy, five preclinical/veterinary, and six technical/pilot reports; 26 were assessed in full text and six from abstracts. Reported outcomes included positive, mixed, and negative directions; heterogeneous endpoints and designs, and the absence of formal risk-of-bias assessment precluded efficacy inference. Low-frequency, low-millitesla regimens recurred, but incomplete and non-equivalent dose reporting precluded identification of an optimal regimen. Contextual bioimpedance evidence supported segmental impedance and pulse-related hemodynamic assessment, not direct measurement of capillary microcirculation. No validated system adapting PEMF from limb bioimpedance for a peripheral-circulation target was identified in the databases searched. The proposed adaptive architecture requires prospective technical and clinical validation.

## 1. Introduction

Peripheral circulation encompasses arterial inflow, venous return, microvascular perfusion, vascular reactivity, and the tissue-level delivery of oxygen and nutrients. Its impairment contributes to peripheral arterial disease (PAD), diabetic foot disease, neuropathy-associated vascular dysfunction, venous disorders, and delayed wound healing. These related processes should not be reduced to vascular tone alone: tone is one physiological determinant within a broader circulatory system. Non-invasive, dosable interventions and objective monitoring methods are therefore of interest for both supervised rehabilitation and future home-based care.

PEMF exposures have been studied across clinical, animal, and technical models using heterogeneous waveforms, frequencies, field strengths, session schedules, and anatomical targets. Reported vascular outcomes include laser Doppler perfusion, photoplethysmographic pulse amplitude, ankle- and toe-brachial indices, vessel diameter, skin perfusion pressure, temperature-based proxies, and angiogenic markers [1,2,3,4]. This diversity provides a broad evidence map but limits direct dose comparison and precludes treating all positive findings as evidence for one common mechanism or protocol.

Bioimpedance and impedance plethysmography provide a complementary measurement track. Localized limb measurements can quantify baseline segmental impedance and its cardiac-synchronous component, from which timing, amplitude, and morphological features may be derived [5,6,7,8,9]. These signals are influenced by blood volume, flow-related erythrocyte orientation, tissue fluid, temperature, geometry, and electrode contact. Accordingly, limb bioimpedance may index pulsatile peripheral hemodynamics but does not directly measure capillary microcirculation and should not be presented as a substitute for laser Doppler flowmetry or other perfusion-reference methods.

The therapeutic and measurement studies have largely developed in parallel. Most PEMF protocols remain fixed during a session, whereas bioimpedance systems are usually evaluated as diagnostic or monitoring instruments. A physiological signal could in principle support adaptive control, but such use requires validated responsiveness, repeatability, artifact control, and a prospectively specified safety-constrained algorithm. The presence of these individual components in separate studies does not by itself validate their integration.

This review therefore aimed to: (i) map original studies of PEMF and peripheral-circulation outcomes; (ii) describe the exposure parameters and outcome measures without inferring a universal optimum; (iii) distinguish direct perfusion evidence from vascular proxies and mechanistic context; (iv) appraise bioimpedance as a candidate monitoring modality while stating what it does and does not measure; and (v) identify precedents and evidence gaps for an adaptive PEMF–bioimpedance system. Meta-analysis was not undertaken because of marked clinical and methodological heterogeneity.

## 2. Search Strategy and Corpus Characterization

### 2.1. Review Type and Reporting

This review was designed as a multistream scoping evidence map and was conducted and reported in accordance with the PRISMA Extension for Scoping Reviews (PRISMA-ScR) [10]; the completed PRISMA-ScR checklist, mapped item by item to the corresponding manuscript sections, is provided as Table S2. Following the JBI guidance for scoping reviews [11], the review question was framed with the Population–Concept–Context (PCC) structure: the population comprised humans, animals, and laboratory models in which peripheral circulation or its surrogates were assessed; the concept comprised pulsed electromagnetic exposure, bioimpedance-based vascular assessment, and adaptive or feedback-controlled stimulation; and the context comprised clinical, preclinical, and engineering settings. A PICO or PECO question was not used because the review maps heterogeneous interventions, measurement methods, and control architectures across clinical, experimental, and engineering evidence rather than comparing a defined intervention or exposure with a comparator for a single outcome. For the main search, screening and charting were conducted by two investigators with AI assistance, but not independently in duplicate. The AI tools were OpenAI ChatGPT/Codex (ChatGPT 5.6) and Anthropic Claude (Claude Opus 5), and their output was advisory. They supported DOI/title deduplication, abstract re-retrieval, organization of the screening tables, consistency checks of the recorded reason codes, and language editing; for each record, they also located the information relevant to the eligibility criteria (exposure type, endpoint, and publication type) and proposed a decision with a reason code and a short rationale. Table S4 records these proposals for every record, together with the evidence-stream assignment or exclusion code adopted by the investigators and, for the 418 s-pass and 99 detailed-assessment records, the decision and its basis; first-pass investigator decisions were not recorded in a separate column. No record was included or excluded on the basis of an AI proposal alone. The first investigator (A.H.) made every first-pass screening and eligibility decision against the criteria in Section 2.3 and undertook the initial charting. During revision, the second investigator (A.B.) re-screened all records whose eligibility had not been established from the indexed title/abstract, using abstracts re-assembled from the database exports or, where missing, re-obtained from OpenAlex or Crossref, with title-level assessment when no abstract was retrievable (Section 2.5), and verified all direct-stream and core-corpus decisions; where the verification differed from the first decision, the record was re-examined by both investigators, and the agreed decision was recorded. Records retrieved by the supplementary IEEE Xplore search added during revision were screened independently in duplicate (Section 2.6).

### 2.2. Search Queries and Flow

Searches were run in Scopus, PubMed, and Web of Science and exported in July 2026. IEEE Xplore, for which access and export were unavailable at that time, was searched during revision as a supplementary source (Section 2.6). Dedicated patent and standards databases, clinical-trial registries, and grey literature were not searched; every absence statement in this review therefore refers only to the four databases searched. Seven structured query families were used; Q6 was split into a narrowly specified Q6a and a broader Q6b to test retrieval sensitivity:

- Q1—PEMF and peripheral circulation (foundational evidence base). Combined the terms “pulsed electromagnetic field*”, “PEMF”, “magnetotherapy”, and “low-frequency magnetic field*” with the descriptors “peripheral circulation”, “peripheral blood flow”, “microcirculation”, “vascular tone”, and “peripheral vascular”.
- Q2—parametric characteristics of PEMF (frequency, intensity, duration). Combined the PEMF terms with the descriptors “frequency”, “intensity”, “dose”, “parameter*”, and “treatment protocol” together with “blood flow”, “vascular”, “circulation”, and “hemodynamic*”.
- Q3—bioimpedance and peripheral hemodynamics. Combined “bioimpedance”, “bioelectrical impedance”, and “electrical impedance” with terms for arterial compliance, pulse wave, vascular resistance, limb, extremity, and peripheral circulation.
- Q4—impedance plethysmography and vasomotor function. Used the specialized terms “impedance plethysmography”, “rheovasography”, and “rheography” together with “vascular tone”, “peripheral resistance”, “arterial tone”, and “vasomotor”.
- Q5—clinical applications of PEMF (target populations and nosologies). Combined the PEMF terms with “peripheral arterial disease”, “Raynaud”, “chronic venous insufficiency”, “diabetic angiopathy”, and “ischemia”, together with “clinical trial”, “randomized”, and “controlled study”.
- Q6a/Q6b—adaptive or feedback-controlled electromagnetic intervention. The narrow formulation (Q6a) required direct co-occurrence of closed-loop/adaptive terminology, electromagnetic therapy, and a physiological feedback signal; the broader formulation (Q6b) expanded stimulation and monitoring synonyms. Q6a returned no records in the three databases, whereas Q6b retrieved a large contextual engineering and neuromodulation stream.
- Q7—PEMF and the endothelial NO-dependent mechanism. Combined the PEMF terms with “endothelial function”, “nitric oxide”, “NO”, “vasodilation”, “smooth muscle”, “vascular”, and “blood vessel*”.

**Figure 1:**
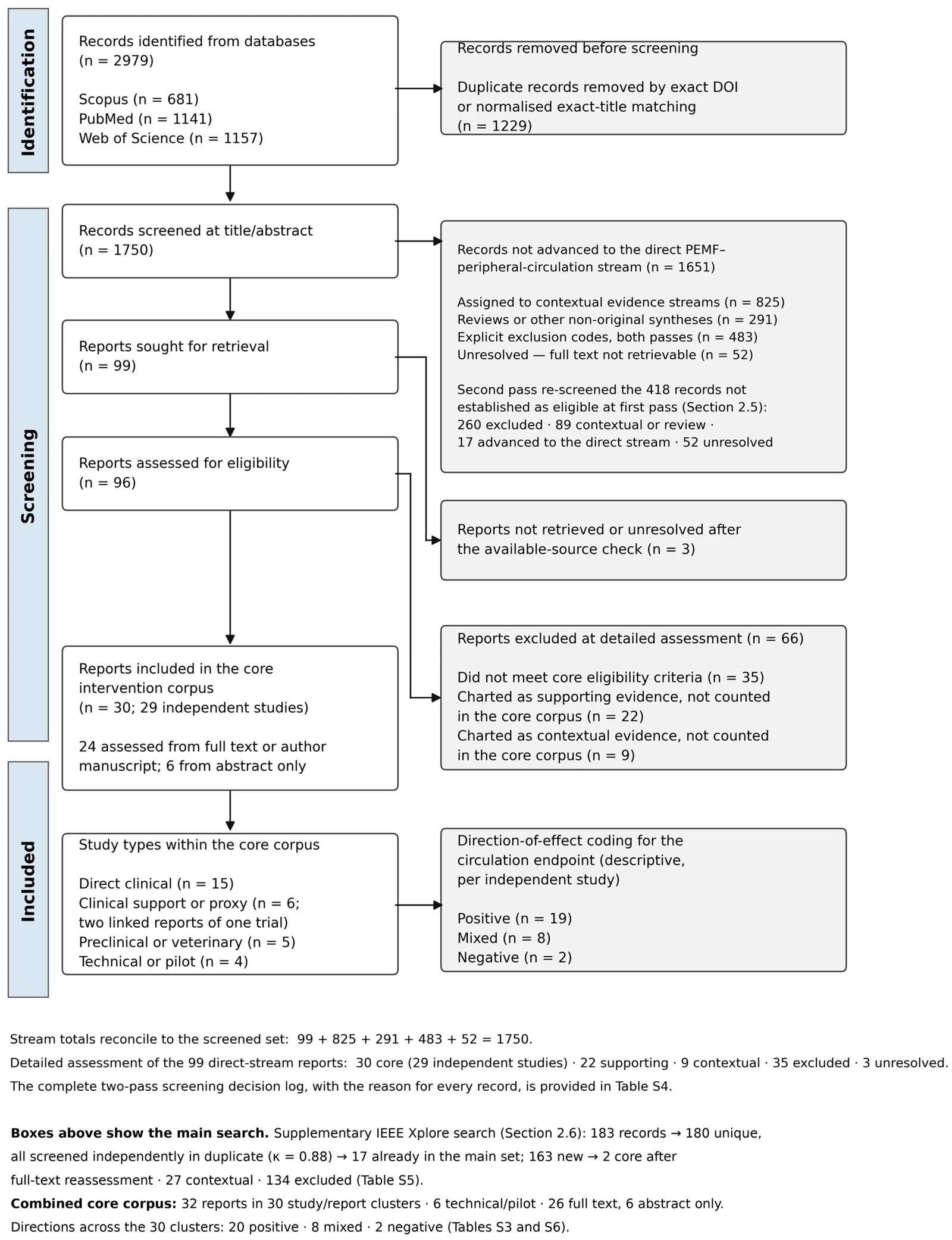
PRISMA flow diagram, adapted for a scoping review. Reporting follows PRISMA-ScR [10]; the PRISMA 2020 flow-diagram template is used because PRISMA-ScR does not publish a separate template. Because the review used a multistream evidence map, records outside the direct PEMF–peripheral-circulation stream were retained as contextual evidence rather than discarded. Of the 825 records assigned to contextual streams after both screening passes, 134 addressed PEMF vascular mechanisms or support, 470 addressed bioimpedance methods (414 limb-vascular and 56 method/support), and 221 were closed-loop electromagnetic precedents. The 418 records for which eligibility could not be established from the indexed title/abstract at first pass were re-screened in a second pass (Section 2.5): 260 then met explicit exclusion codes, 106 were assigned to evidence streams (17 direct, 81 bioimpedance or mechanism contextual, 8 review or closed-loop), and 52 remained unresolved because full text could not be retrieved. Screening was conducted in two sequential investigator passes with AI-assisted triage: the first-pass screening and initial charting were undertaken by one investigator, and the second-pass re-screening and final direct-stream/core verification by the second investigator; screening was not performed independently in duplicate, and the review protocol was not registered. The complete screening record is provided in Table S4. The supplementary IEEE Xplore search (Section 2.6) is summarized beneath the diagram and reported in full in Table S5. Boxes in the diagram show the main search; the lines beneath it give the final dispositions of the supplementary IEEE records after full-text reassessment and the totals for the combined core corpus. The combined core corpus contains 32 reports grouped into 30 study/report clusters under the conservative Jo grouping convention (Section 2.6; Tables S3 and S5).

The searches yielded 2979 records: 681 from Scopus, 1141 from PubMed, and 1157 from Web of Science. Exact DOI or normalized exact-title matching removed 1229 duplicates, leaving 1750 unique records for title/abstract mapping. After both screening passes (Section 2.5), 99 records were assigned to the direct PEMF–peripheral-circulation stream; 825 to contextual streams (134 PEMF vascular-mechanism/support records, 470 bioimpedance records—414 limb-vascular and 56 method/support—and 221 closed-loop electromagnetic precedents); and 291 to reviews or other non-original syntheses used only for context and citation chasing. Of the remaining 535 records, 483 met explicit exclusion codes (223 at first pass and 260 at or after the second pass) and 52 remained unresolved because eligibility could not be established from the indexed record and full text could not be retrieved; these were retained as unresolved rather than treated as excluded. Detailed assessment of the 99 direct-stream records classified 30 reports (29 independent studies) as core, 22 as supporting, 9 as contextual, 35 as excluded, and 3 as unresolved after the available source check. Figure 1 and Table 1 report the auditable flow and query yields; the complete deduplicated screening record, with the stream assignment and exclusion reason for every record, is provided as Table S4. These figures describe the main search flow; two additional technical reports from IEEE bring the combined corpus to 32 reports (Section 2.6).

### 2.3. Eligibility Criteria

Core intervention studies were eligible when they were original peer-reviewed reports published in 2010–2026, used a pulsed electromagnetic exposure, and reported at least one quantitative peripheral-circulation, vascular-response, ischemic-limb, or parameter-sensitive pulse/hemodynamic endpoint. Direct clinical outcomes were distinguished from vascular-function proxies, preclinical models, and technical or in vitro studies. Reviews, non-pulsed exposures, records without an interpretable vascular endpoint, and non-original reports were excluded from the core intervention synthesis but could be cited as contextual evidence. Abstract-only reports were retained when intervention and outcome information was sufficient for limited charting; missing details were reported as not available rather than inferred.

The 2010–2026 interval was selected pragmatically to focus the core intervention map on recent literature within a manageable review scope. The 2010 cut-off was not based on a demonstrated technological breakpoint or an assumption that earlier systems had ceased to be used. Earlier publications were retained as historical or architectural context when identified, but were not included in the core intervention count. This date restriction limits historical completeness and may omit relevant earlier intervention evidence.

Technical bioimpedance and adaptive-control publications were mapped as separate contextual streams because they address measurement feasibility or architecture rather than the efficacy of PEMF for peripheral circulation. For reproducibility, the contextual mapping and charting were handled in two stages. At the broad mapping stage, records retrieved by the bioimpedance queries were assigned to a limb/peripheral bioimpedance stream when the indexed title or abstract suggested possible peripheral or vascular relevance, or to a method/support stream when they addressed impedance-cardiography, instrumentation, or signal processing without a limb-vascular application; these stream labels are retrieval and evidence-mapping categories rather than final eligibility judgements. For the charted technical evidence used in Section 3.5 and Section 3.6, a stricter exemplar criterion was then applied: direct relevance to peripheral pulse-synchronous or vascular/hemodynamic impedance measurement. Records in which bioimpedance was used solely for body composition, nutritional status, hydration or dialysis fluid status, respiration, or cellular assays were therefore not charted as vascular exemplars, even if they remained traceable within the broad contextual mapping in Table S4. The closed-loop stream included original or technical reports of adaptive, feedback-controlled, or closed-loop electromagnetic or magnetic stimulation; closed-loop control of non-electromagnetic interventions was excluded. Section 3.5, Section 3.6 and Section 3.7 chart all records meeting the stated exemplar criteria rather than a representative sample; contextual records not meeting those criteria remain in the stream counts and in Table S4 but were not charted individually. Foundational sources outside the 2010–2026 window, including pulse-wave methods and the 2009 cardiac-synchronized rheovasography-guided PEMF antecedent [12,13,14,15,16], were used only for historical or conceptual context and were not counted in the intervention corpus.

### 2.4. Data-Charting Framework

For each of the 32 core intervention reports, the charting template recorded study design, population or model, sample size, exposure modality and parameters, comparator, anatomical target, circulation endpoint, measurement method, timing, direction of result, source availability, and key limitations, together with quality characteristics (randomization, control condition, and blinding as reported); the completed charting table for all 32 reports is provided as Table S3. Direction was classified as positive, mixed, or negative for the circulation endpoint—not for pain, wound healing, or other co-primary outcomes. Technical records were charted separately for probing frequency, current, electrode configuration, sampling, signal features, and validation method.

A descriptive source-availability sensitivity analysis repeated the evidence map after removing reports assessed from abstracts only. Evidence streams were retained separately, and linked reports were counted once at the study level. If an abstract-only report had a full-text companion, the study remained represented through the accessible report. This analysis assessed the distribution and coverage of the charted evidence, without estimating a pooled treatment effect. Report-level exclusions and stream-specific results are given in Table S6. Complete published conference reports were treated as full-text sources even when abbreviated. For the Jo pair, study-level analyses use one potentially overlapping report family as a conservative counting convention; a separate-report sensitivity count is also reported. This does not establish that the biological samples were identical.

Query yield should not be interpreted as evidence density. Broad wording in Q3, Q6b, and Q7 produced many contextual records, whereas the narrow Q6a formulation produced none. Eligibility and evidence role were therefore determined at record level after deduplication rather than from query membership.

### 2.5. Second-Pass Screening of Records with Unestablished Eligibility

At first-pass title/abstract screening, 418 of the 1750 unique records could not be established as eligible from the indexed record (277 with unconfirmed pulsed exposure, 140 with uncertain vascular–bioimpedance relevance, and one with insufficient indexed information) and were initially not advanced. Because failure to confirm eligibility at the title/abstract stage is not equivalent to confirmation of ineligibility, all 418 records were re-screened during revision in a structured second pass. Abstracts were re-assembled from the original database exports for 308 records and independently re-obtained from OpenAlex or Crossref for a further 73 records; 37 records for which no abstract could be retrieved were assessed at title level, with exclusion assigned only where the title was decisive. The second pass applied two operational rules for the revision-stage reassessment: an exclusion code was assigned only when the abstract—or, for the 37 records without a retrievable abstract, the title—positively established the exclusion reason, and any record for which a pulsed exposure and a peripheral vascular endpoint were both plausible was advanced to full-text assessment rather than excluded.

Of the 418 re-screened records, 260 met explicit exclusion codes, 81 were assigned to the bioimpedance or mechanism contextual streams, 8 were assigned to the review or closed-loop streams, 17 were advanced to the direct PEMF–peripheral-circulation stream for detailed assessment, and 52 remained unresolved because eligibility could not be settled from the available record and full text could not be retrieved; unresolved records were retained as unresolved rather than excluded. Detailed assessment of the 17 advanced records added 10 reports to the core corpus (two of them after full-text retrieval during the revision), and excluded seven for an ineligible vascular endpoint or publication type: five decisions were based on full-text assessment and two on abstracts, as documented in Table S4. One further record, initially unresolved, was resolved by full-text retrieval during the revision and excluded because its transcerebral intervention combined alternating and static magnetic fields rather than a pulsed exposure [17]; it is counted among the 260 s-pass exclusions. The second pass was performed with AI assistance under the rules above, and every stream-level, exclusion, and core decision was verified by the investigators; the complete second-pass decision log, including the decision basis and reason for every record, is provided in Table S4. The re-screening also identified two core reports that appear to describe the same trial: they share the device, treatment protocol, and participant age profile and report 55 and 54 completers (Kóródi and Rikk reports, respectively). Because participant identity could not be confirmed, they are conservatively counted as linked reports of a single study in all study-level tallies [18,19].

### 2.6. Supplementary IEEE Xplore Search

Because IEEE Xplore could not be searched at the original search date, it was searched during revision, on 21 September 2026, to test the engineering-facing absence statements. Command Search was applied to all metadata without date, language, or content-type limits, using four structured queries that mirror the concepts of the main strategy (Table S1): PEMF or magnetic-stimulation terms combined with peripheral-circulation terms (IEEE-Q1), with bioimpedance and impedance-plethysmography terms (IEEE-Q2), and with closed-loop or adaptive-control terms (IEEE-Q3), and bioimpedance terms combined with closed-loop, circulation, and stimulation terms irrespective of the stimulation modality (IEEE-Q4). A fifth, targeted query on magnetic-stimulation artifacts and electromagnetic compatibility was used only to identify technical literature and did not contribute to the flow.

The four queries retrieved 183 records (IEEE-Q1, 40; IEEE-Q2, 29; IEEE-Q3, 114; IEEE-Q4, 0), or 180 after within-database deduplication. Seventeen were already present in the main set as broad contextual mappings, leaving 163 new records. Two investigators (A.H. and A.B.) independently screened all 180 titles and abstracts, blinded to each other’s decisions. Their original ratings agreed for 172 records (95.6%; Cohen’s κ = 0.88, approximate 95% CI 0.80–0.96). Eight disagreements and one record rated uncertain by both were resolved by consensus; the latter was resolved after full-text inspection. The original ratings and initial consensus are retained in Table S5. Full-text reassessment included two microvascular-model reports (IEEE-018 and IEEE-019 [20,21]) as core technical/in vitro evidence because both used pulsed magnetic exposure and reported quantitative RBC-flow outcomes; in vitro status alone is not an exclusion under Section 2.3. The journal article and complete one-page conference report contain different numerical velocities, sedimentation observations, and micrographs. Their identical title, authors, model and exposure apparatus suggest related work, but sample overlap or independence is not established. They are retained as two reports and conservatively grouped into one Jo study/report cluster for descriptive study-level counting, with a separate-report sensitivity count. This analytic convention does not classify them as identical experiments or merge them with the stenosed-channel report that used human RBCs (Section 3.4.6). Among the 163 new records, 2 are core, 27 contextual, and 134 excluded; none remains unresolved. Table S5 retains original human ratings and initial consensus separately from the revision decisions. Broad contextual mapping in Table S4 is distinguished from final exemplar eligibility in Table S5. The contextual records include on-line impedance monitoring during pulsed magnetic therapy and proposed biotechnical feedback for adapting magnetotherapy (Section 3.7.6).

## 3. Results of the Literature Mapping

Results are presented in three linked layers. Section 3.1, Section 3.2, Section 3.3 and Section 3.4 describe the core intervention corpus of 32 reports (30 study/report clusters under the Jo grouping convention) and its exposure, endpoint, and population heterogeneity. Section 3.5 and Section 3.6 summarize targeted technical evidence on limb bioimpedance and impedance plethysmography. Section 3.7 maps contextual precedents for adaptive control. Keeping these layers separate prevents technical feasibility studies from being counted as therapeutic-effect evidence.

No formal risk-of-bias instrument was applied. Outcome-direction counts are descriptive and give equal weight to studies with very different designs; they do not estimate efficacy and are vulnerable to publication bias, small-study effects, possible cohort overlap, and incomplete reporting. They are used to characterize the map, not to produce a pooled treatment effect. Study-level design characteristics are summarized in Section 3.4.8 and, per report, in Table S3. These characteristics are not a formal risk-of-bias score. Interpretation is restricted by evidence stream, source availability, controls, blinding, co-interventions, and endpoint relevance; neither outcome-direction counts nor frequently used exposure settings establish therapeutic efficacy.

### 3.1. Action of PEMF on Peripheral Circulation: Overview of the Evidence Base

The combined core comprised 32 reports: 30 reports representing 29 established studies from the main search and two Jo technical reports from IEEE. Conservatively grouping the potentially overlapping Jo pair yields 30 study/report clusters, not 30 verified independent experiments. Fifteen reports were direct clinical, six used clinical vascular-function or cardiovascular proxies (including two reports conservatively linked as one trial), five were preclinical/veterinary, and six were technical/pilot. Twenty-six reports were assessed from complete published texts or author manuscripts and six from abstracts. Across the 30 clusters, descriptive outcome directions were 20 positive, eight mixed, and two negative. These unweighted counts do not establish clinical efficacy or statistical significance; endpoints and designs differed, and no formal risk-of-bias assessment was performed.

Restricting the map to complete available texts retained 26 reports grouped into 25 study/report clusters: 12 direct clinical, five clinical support/proxy, four preclinical/veterinary, and five technical/pilot reports. The abstract-only Kóródi report was removed, while the linked study remained represented by Rikk. Both complete Jo publications were retained; the conference report remains limited by abbreviated reporting. With the conservative Jo grouping, directions changed from 20 positive, eight mixed, and two negative across 30 clusters to 18 positive, six mixed, and one negative across 25 full-text clusters. Treating the Jo reports as separate units instead gives 31 total units (21 positive, eight mixed, two negative) and 26 full-text units (19 positive, six mixed, one negative). Neither convention establishes the independence of the Jo samples. Direct clinical full-text findings remained nine positive, two mixed, and one negative among 12 studies. Evidence coverage narrowed, and these descriptive analyses do not establish efficacy or robustness of a dosing recommendation (Table S6).

The full-text-only analysis excluded six abstract-only reports and reduced the number of study/report clusters from 30 to 25 (Table S6). Rikk (2013) [19] continues to represent the linked study that also includes the Kóródi (2016) [18] report. Report identifiers and references are provided in Tables S3 and S6. Claims relying on removed reports remain abstract-supported observations rather than independently confirmed full-text findings; this analysis does not establish the robustness of individual dose recommendations.

The direct clinical evidence was concentrated in diabetic foot disease, diabetic peripheral neuropathy, and Fontaine stage II PAD. Objective measures included LDF, PPG, ABI, TBI, skin perfusion pressure, small-vessel diameter or velocity, and claudication performance [22,23,24,25,26,27,28,29,30]. These endpoints capture different levels of the circulation and are not interchangeable.

In diabetic foot ulcer, Alghadier et al. reported improvements in LDF perfusion, PPG amplitude, TBI, and ABPI after an eight-week PEMF regimen, with persistence at follow-up [24]. Kwan et al. reported improved capillary velocity and diameter in a small randomized pilot [22]. Both support a possible distal-perfusion effect but are limited by small groups; the former lacked a sham-PEMF arm, and the latter was available only as an abstract.

More rigorous or acute studies did not yield uniformly positive results. In the multicenter sham-controlled trial of pulsed radiofrequency electromagnetic therapy for painful diabetic neuropathy, the between-group change in skin perfusion pressure was not significant (*p* = 0.670) [25]. Sun et al. found a mixed acute response across small-vessel and cutaneous LDF measures at 12 Hz and 0.5 mT [23], while Biermann et al. reported no clear cutaneous-flow benefit in a small within-participant study [31].

Three small PAD studies reported improved ABI and walking performance [26,27,29]; two of them used 15 Hz at approximately 2 mT [26,29], whereas the third did not report the frequency or field strength [27]. Interpretation is constrained by absent sham conditions, unclear or non-random allocation, pharmacological or exercise co-interventions, and possible overlap between closely related publications. These studies justify confirmatory trials but do not establish a definitive dose.

Two additional diabetes-related rehabilitation studies reported improvements in LDF-derived foot perfusion or ABI [28,30]. One used a crossover sequence with interval training and no washout [28]; the other compared PEMF with shock-wave therapy and reported device intensity only as a percentage [30]. Both were positive but cannot isolate a precise PEMF dose–response relationship. Five further direct clinical studies were added by the second-pass screening (Section 2.5). A randomized controlled study of high-intensity pulsed magnetotherapy in chemotherapy-induced peripheral polyneuropathy (*n* = 60) reported significant improvement of extremity LDF microcirculation with normalization of arteriolar tone and reduction in venous stasis [32]. A placebo-controlled study of impulse magnetotherapy in stage I–II lower-limb lymphedema (*n* = 59) reported improvement of all LDF-derived components of the microcirculatory stream [33]. A study of a running impulse magnetic field applied along the limb in children with diabetic polyneuropathy (*n* = 45) reported improved LDF microcirculation when the field-run velocity matched nerve conduction velocity [34]. A randomized study of a rehabilitation complex including explicitly pulsed magnetotherapy (10 mT, 5 ms pulses, solenoid inductors on the lower extremities) in post-thrombotic syndrome (*n* = 60) reported significant LDF flowmotion improvement versus a compression-plus-venotonics control [35]. A randomized study of complex physical therapy including low-frequency (10–50 Hz, 10 mT) magnetotherapy of the calf in Fontaine stage II PAD (*n* = 47) reported improved claudication distance in both arms without a significant ankle-brachial-index change [36]. One related Russian rehabilitation report—on arterial hypertension—was resolved at full text during the revision: the intervention combined alternating and static transcerebral magnetic fields rather than a pulsed exposure, and the report is therefore excluded from the core corpus [17].

The clinical proxy/support reports (six, representing five studies) broadened, but did not directly answer, the peripheral-perfusion question. Stewart et al. reported improved brachial flow-mediated dilation and plasma NO in hypertension [4]. Rikk et al. reported reduced systolic and pulse blood pressure without change in arterial stiffness [19], and Kóródi et al. reported mixed changes in temperature and cardiovascular measures [18]; these two reports appear to derive from the same trial (Rikk et al. describe a double-blind, sham-controlled design; both reports use the same device and protocol, with a similar participant age profile and 54 and 55 completers, respectively); because participant identity could not be confirmed, they are conservatively charted as linked reports of one trial and counted once in study-level tallies. Three proxy reports were added by the second pass: a one-arm pilot of a biorhythmically configured impulse electromagnetic field in multiple-organ-dysfunction patients reported an approximately 25% increase in sublingual microvascular flow index with parallel hemodynamic stabilization (*n* = 10) [37]; sacral-root functional magnetic stimulation in spinal-cord injury increased ischial skin perfusion during stimulation (*n* = 14) [38]; and 20 Hz radial-nerve magnetic stimulation in healthy adults increased cephalic venous blood flow after a single session (*n* = 25) [39]. Proxy outcomes were therefore not pooled with direct limb-perfusion endpoints.

Preclinical and veterinary studies generally supported biological responsiveness but had limited clinical generalisability. Hindlimb-ischemia models reported improved temperature ratios, capillary density, or perfusion [2,40,41]; a rabbit study of pulsed magnetic stimulation of the carotid sinus reported an intensity-dependent blood-pressure reduction [42], and a small sham-controlled equine study found immediate vessel-diameter changes but mostly null thermography [43]. Technical studies used substantially higher fields and acute PPG or microfluidic outcomes [44,45,46], and one study characterized magnetic-stimulation-related artifacts in peripheral hemodynamic (photoplethysmographic and near-infrared) signals [47]; these are engineering observations, not clinical dose guidance.

- Outcome direction in the core corpus was descriptive and heterogeneous (counts above).
- The coexistence of positive, mixed, and null results is more informative than a single percentage of success. It shows that response depends on study design, biological context, exposure specification, and endpoint selection; it also cautions against explaining every negative result after the fact as an incorrect dose.
- Low-frequency protocols around 10–30 Hz and low-mT field strengths recurred in several positive clinical and preclinical studies, but positive results also occurred under incompletely reported regimens, while null or mixed results occurred both inside and outside that range.
- Accordingly, the evidence supports parameter-sensitive investigation rather than an established universal optimum. A valid dose comparison must report waveform, frequency, field distribution at the tissue, dB/dt or induced electric field where feasible, pulse duration, session schedule, target anatomy, and comparator.
- None of the 32 core intervention reports used limb bioimpedance pulse-wave features as a real-time feedback signal to alter PEMF parameters. Response monitoring relied mainly on LDF, PPG, pressure indices, ultrasound, temperature, or functional outcomes.
- The two Jo 2018 reports [20,21] (Table S3, IEEE-018 and IEEE-019) used rabbit blood in straight PDMS microchannels and a 1 Hz pulsed magnetic system with a maximum field of 0.27 T. The journal reported RBC velocities of 71.4 before and 76.8 µm/s after exposure; the conference report gave 15.5 and 19.2 µm/s. The journal described a 3 min exposure and 102 µs pulse duration including three micropulses; the conference described a 0.102 ms transition time but did not specify exposure duration. Both reported slower sedimentation after exposure. Biological sample sizes, replicate counts and inferential statistics were not reported. These are descriptive positive laboratory findings, not demonstrated improvements in patient perfusion, oxygen delivery or treatment efficacy.

This absence defines the central translational gap: intervention effects and bioimpedance measurement capabilities have each been studied, but their real-time integration for peripheral-circulation therapy has not been validated.

### 3.2. The Parametric Landscape of PEMF

The core studies used heterogeneous exposure families rather than a common, directly comparable dose. Frequency alone is insufficient because waveform, field amplitude, coil geometry, rise time, duty cycle, and treatment schedule determine the induced exposure. The map therefore describes recurrent regimes and uncertainties instead of ranking protocols by efficacy.

#### 3.2.1. Frequency Range

**Figure 2:**
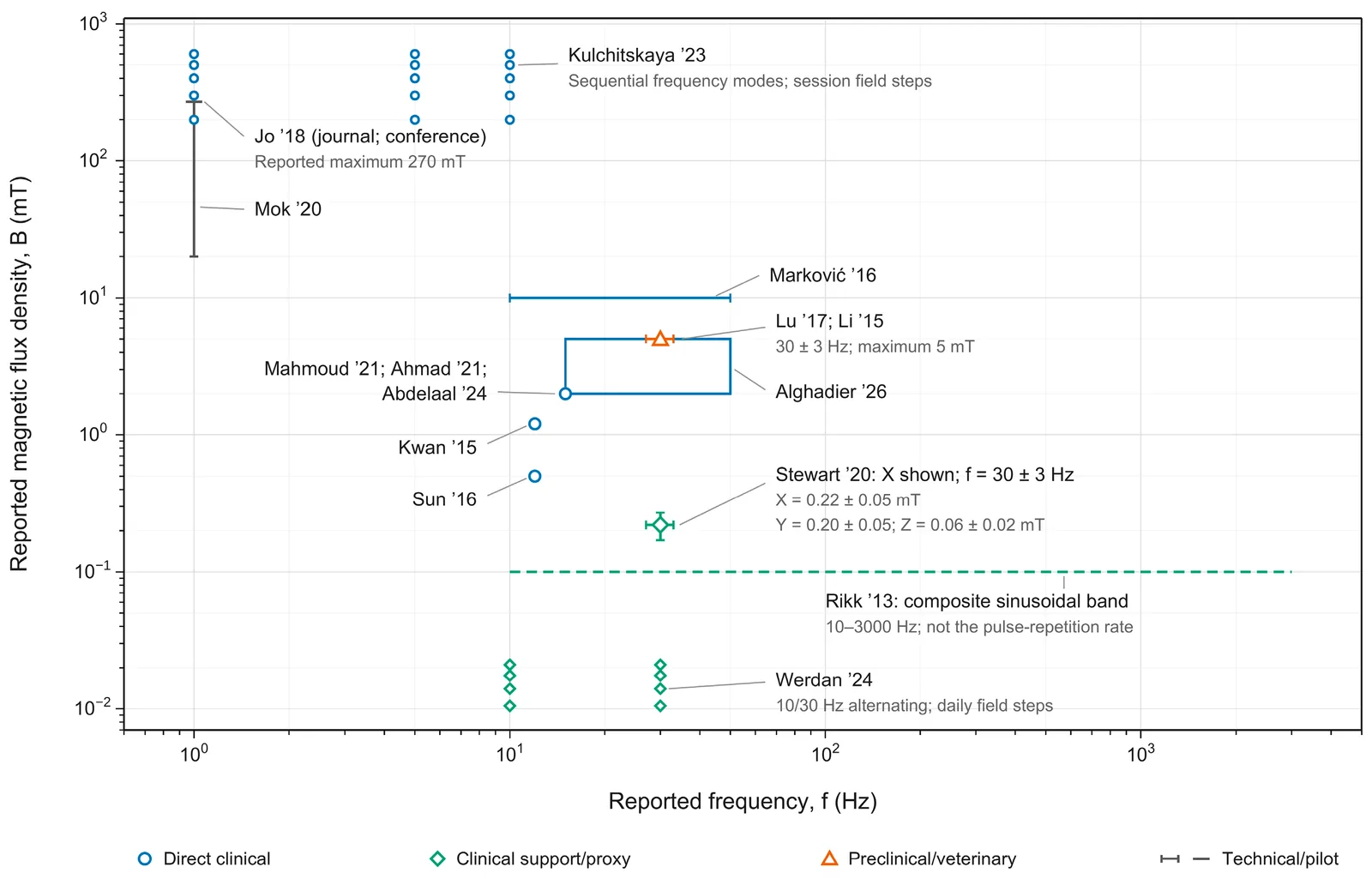
Reported frequency and magnetic flux-density information for 16 of the 32 core intervention reports. Symbols indicate reported discrete or nominal settings; lines and the unfilled envelope indicate reported bounds or variation, not confidence intervals or evidence that every intermediate value or combination was tested. For Stewart, the X component is plotted at 30 ± 3 Hz and 0.22 ± 0.05 mT; the Y and Z components are given alongside it, and no resultant field is inferred. Lu and Li report 30 ± 3 Hz. The ± values are reproduced as reported without assigning a statistical interpretation. The two Jo 2018 [20,21] reports share a short horizontal tick at 1 Hz and 270 mT, coincident with the upper endpoint of Mok’s unchanged 20–270 mT range. Jo’s value is the reported system maximum (5 mm from the coil in the journal report; measurement location not reported in the conference report), not a verified uniform field throughout the blood sample. The reports are conservatively grouped as one family, with exact sample overlap unconfirmed. Kulchitskaya’s points show the sequential 1, 5 and 10 Hz modes at the session field levels of 200, 300, 400, 500 and 600 mT. Werdan’s alternating 10/30 Hz bands are shown at the reported daily levels of 0.0105, 0.014, 0.0175 and 0.021 mT. Rikk’s dashed 10–3000 Hz interval is the composite waveform’s sinusoidal component band, plotted at the reported system maximum of 0.1 mT; it is neither the pulse-repetition rate nor the amplitude of individual spectral components. Ranges beginning at zero are represented at their reported maximum on the logarithmic axis. Co-located reports share a symbol, and one report may contribute multiple settings. Frequency definitions, field quantities and measurement locations, waveforms and dosimetry differ; shared axes do not establish exposure equivalence. The 16 reports not represented on these axes are listed in Table 2; report citations and charting details are in Table S3. The map describes reporting and comparability without estimating efficacy or an optimal regimen. Report labels in the figure correspond to the following references: Stewart ’20 [4]; Rikk ’13 [19]; Jo ’18 [20,21]; Kwan ’15 [22]; Sun ’16 [23]; Alghadier ’26 [24]; Mahmoud ’21 [26]; Abdelaal ’24 [28]; Ahmad ’21 [29]; Kulchitskaya ’23 [32]; Marković ’16 [36]; Werdan ’24 [37]; Lu ’17 [40]; Li ’15 [41]; Mok ’20 [44].

Exposure parameters were reported in several non-equivalent ways across the complete corpus (Figure 2 and Table 2):

- Sixteen reports specified frequency and magnetic flux-density information suitable for the shared axes: most reported 10–50 Hz with microtesla-to-millitesla fields, with one high-intensity clinical protocol at 200–600 mT, Mok’s technical report at 20–270 mT, and the two Jo technical reports at 1 Hz with a stated system maximum of 270 mT. These are reporting coordinates, not equivalent absorbed doses;
- Six reports provided an incomplete frequency–flux-density pair (one or both values not reported), including two high-field technical studies at approximately 0.4 and 0.8 T;
- The remaining ten reports used dose metrics that cannot be expressed on a shared frequency–flux-density map: one burst-radiofrequency study (27.12 MHz carrier, dosed by forward power and duty cycle), four magnetic nerve- or motor-stimulation studies (three dosed relative to motor threshold and one without reported parameters), and five reports with no recoverable exposure parameters.

Within the low-frequency clinical subset, 15 Hz appeared repeatedly in PAD and diabetic rehabilitation studies [26,28,29], while 10–12 Hz and 15–50 Hz appeared in other diabetes-related studies [22,23,24,30]. The recurrence of a value does not demonstrate optimality, particularly where studies are small, protocols differ, and independent replication is limited.

The most consistent 15 Hz findings involved ABI or walking outcomes in PAD, typically with approximately 2 mT and sessions of 24–60 min [26,28,29]. These studies were positive but included co-interventions or lacked sham control. Their results identify a reasonable candidate regimen for dose-finding, not a clinical standard.

At 12 Hz, a diabetic-foot pilot reported improved capillary measures [22], whereas an acute study at 12 Hz and 0.5 mT produced mixed vascular findings [23]. At 30 ± 3 Hz, hindlimb-ischemia models reported improved perfusion or neovascularisation [40,41]. These differences illustrate the strong dependence on model and endpoint.

Adjacent mechanistic and regenerative studies use additional frequency bands and tissue targets [26,48,49,50,51,52,53,54,55,56,57,58]. They can inform hypotheses about cellular responsiveness but were not counted as direct evidence of improved peripheral circulation unless they met the core eligibility criteria.

The 27.12 MHz pulsed-radiofrequency regimen requires separate interpretation. In the rigorous sham-controlled diabetic-neuropathy trial, the skin-perfusion-pressure endpoint did not differ between groups [25]. Mechanistic claims that this carrier or burst pattern is specifically “resonant” with calmodulin remain hypotheses and should not be treated as clinically verified dose selection.

#### 3.2.2. Field Intensity and Limits of Cross-Study Dose Comparison

The available data do not establish an inverted-U biological window for limb circulation. Apparent clusters may reflect device families, selected models, and publication patterns rather than a fitted dose–response relation (Table 3).

- Clinical low-frequency studies most often used low-mT fields, but not all were positive, and several reported insufficient physical dosimetry;
- Very-low-field BEMER-type studies used different waveform systems and produced mixed or endpoint-dependent findings [18,19,31,43];
- High-field technical studies assessed acute PPG or in vitro hemodynamic responses and cannot define a therapeutic window for patients [44,45,46];
- Radiofrequency and low-frequency exposures cannot be compared by magnetic flux density alone because the induced electric field and duty cycle differ by orders of magnitude.

A biological-window hypothesis remains plausible but untested in this corpus. Demonstration would require a prospective within-model dose–response study holding waveform, coil geometry, anatomy, schedule, and endpoint constant while varying one exposure dimension. Cross-study interpolation cannot substitute for that experiment.

Static-field and high-intensity regenerative studies are useful mechanistic context but fall outside the core pulsed-intervention question. Their inclusion in the same dose curve would obscure rather than clarify the evidence.

Dose reporting should therefore include field distribution, waveform, rise/fall time, dB/dt, and induced electric field where feasible. Magnetic flux density alone is particularly inadequate for comparisons spanning low frequency, burst radiofrequency, and high-field technical stimulation.

#### 3.2.3. Pulse Shape, Session Duration, and Course Length

Waveform reporting was incomplete in several core studies. A rectangular 4.5 ms pulse was reported in closely related PAD protocols [26,29], but internal inconsistencies in reported timing require source-level caution. Other studies reported asymmetric waveforms, burst radiofrequency, or only a device setting. The evidence does not support declaring one pulse shape standard.

Clinical schedules ranged from acute single sessions to repeated treatment over 18 weeks. Common schedules included 24–60 min per session and courses of 8–12 weeks, while the radiofrequency trial used 30 min twice daily for four months [25]. Because populations and co-interventions differed, neither a minimum effective course nor an optimal session duration can be inferred.

#### 3.2.4. What Can Be Concluded from the Parameter Map

The defensible conclusion is a set of candidate starting ranges, not an optimum. Low-frequency, low-mT protocols around 10–30 Hz recur in clinical studies of PAD and diabetes, especially near 15 Hz and approximately 2 mT, but the evidence is insufficient for universal recommendations. Future trials should compare pre-specified doses within the same population and endpoint, include sham control, report field dosimetry, and separate acute vasomotor effects from long-term vascular remodeling.

Systematic reviews in adjacent neurological, musculoskeletal, and regenerative fields report broad and partly overlapping parameter ranges [48,59]. These contextual syntheses reinforce the need for target-specific dosimetry rather than validating a peripheral-circulation optimum.

### 3.3. Molecular Mechanisms of the Vascular Response

**Figure 3:**
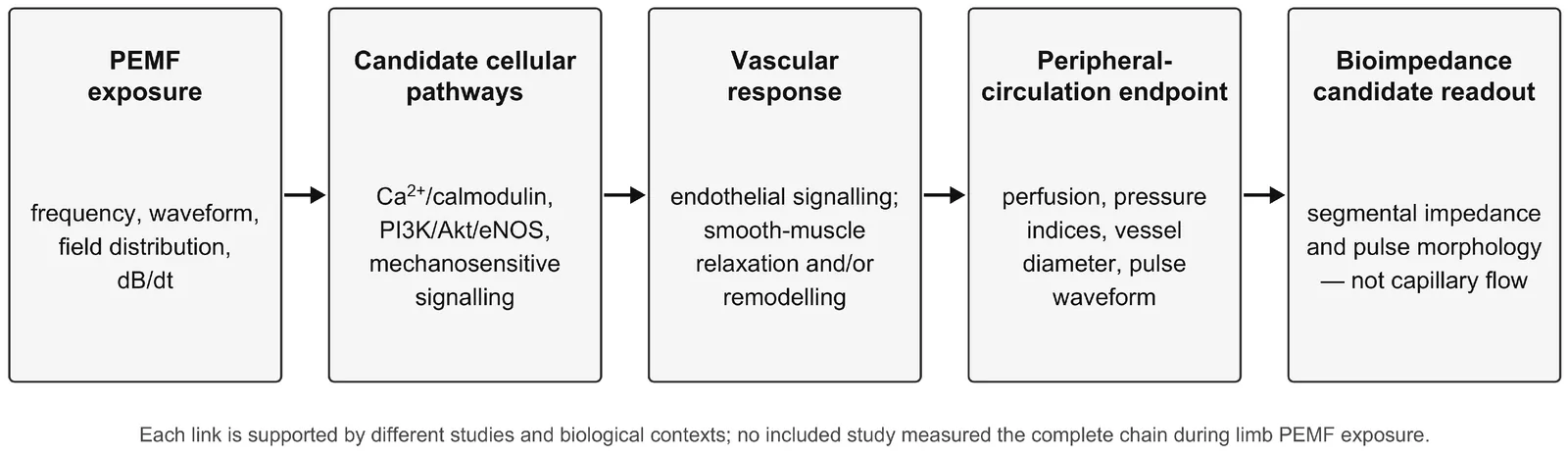
Mechanistic hypothesis linking PEMF exposure to a measurable peripheral-circulation response. Evidence for the individual links comes from different studies and biological contexts; no included study measured the complete chain during limb PEMF exposure. Bioimpedance is shown as a candidate segmental readout, not a direct measure of capillary microcirculation.

The mechanistic literature provides a plausible set of pathways by which some PEMF exposures could alter endothelial and vascular responses. Because the relevant links arise from different tissues, models, and exposure regimes, Figure 3 presents a hypothesis map rather than a single experimentally demonstrated cascade. The candidate pathways below are drawn from different tissues, species, and exposure conditions, including non-identical pulsed regimens; their proposed integration should therefore be read as biologically plausible but not as a validated pathway for any specific clinical PEMF protocol, and the complete chain has not been demonstrated within a closed measurement–stimulation loop.

#### 3.3.1. Candidate Calcium–Calmodulin Pathways

Time-varying electromagnetic exposure can induce electric fields and may influence calcium-dependent and mechanosensitive signalling. Calcium–calmodulin interactions and downstream eNOS regulation are recurrent mechanistic hypotheses [48]. Evidence from non-peripheral and postoperative models is informative but does not demonstrate that a specific carrier frequency or burst pattern is selectively resonant with calmodulin in the human limb.

#### 3.3.2. eNOS-Related Observations in Preclinical PEMF Studies

General vascular physiology provides the rationale for investigating calcium-dependent eNOS regulation and NO production. The studies below assessed selected signalling or vascular outcomes in different preclinical models; they did not demonstrate this entire sequence during peripheral PEMF treatment. In the work of Ma et al., in a model of myocardial ischemia/reperfusion, activation of the p-Akt/p-eNOS pathway was shown, accompanied by increased mobilization of endothelial progenitor cells (EPCs) and improved left-ventricular function [60]. Lu et al., in a model of PAD in hypertensive rats, demonstrated that combining PEMF with therapeutic ultrasound produces a maximal increase in perfusion and capillary density with a simultaneous reduction in the apoptotic index (TUNEL) [40]. The combined-intervention result does not isolate a PEMF-only effect. In the study of Hao et al. (2014), in a model of myocardial infarction, eNOS activation and enhanced neovascularisation were confirmed [61], and Li et al. (2015) [41] showed that PEMF at 30 ± 3 Hz/5 mT/8 min × 4 cycles per day for 14 days accelerates postnatal neovascularisation through eNOS activation and EPC mobilization [41].

PI3K/Akt/eNOS signalling has been implicated in several preclinical models [40,41,60,61]. These studies support biological plausibility, but pathway blockade in one model does not establish that the same pathway explains every clinical circulation response or every PEMF regimen.

Reported eNOS responses are context-dependent across injury, inflammatory, and ischemic models [40,41,58,60,61]. This heterogeneity argues against treating eNOS as a universal scalar mediator and supports direct measurement of both vascular outcomes and mechanistic biomarkers in future trials.

#### 3.3.3. NO–cGMP Vascular Physiology and the Proposed Impedance Link

NO activates soluble guanylate cyclase in vascular smooth muscle and can promote cGMP-dependent relaxation. Stewart et al. reported an increase in plasma NO together with improved flow-mediated dilation in hypertension [4]. Pharmacological blockade data from cerebral microcirculation provide mechanistic support in that model [1], but extrapolation to limb protocols remains indirect.

**Figure 4:**
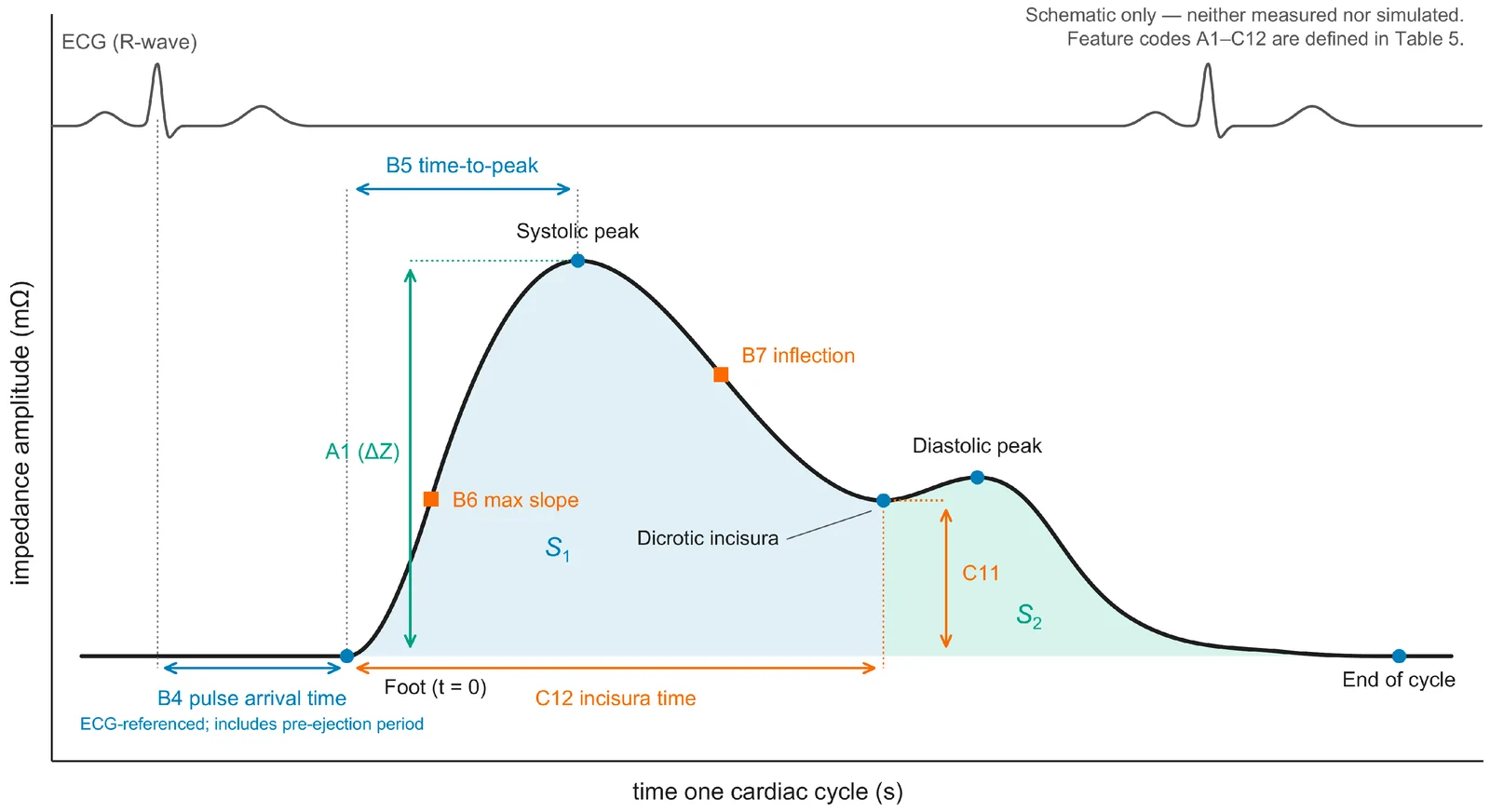
Schematic impedance pulse waveform with the fiducial points and the feature codes defined in Table 5. The waveform is a schematic constructed for definition purposes only; it is neither measured nor simulated data, and the axis units identify the plotted quantities without implying a scale. B4 is referenced to the ECG R-wave and therefore includes the cardiac pre-ejection period. Shaded areas S_1_ and S_2_ denote the systolic and post-incisura portions of the pulse area used by the area index. These candidate monitoring variables require repeatability, responsiveness, artifact, and reference-method validation before any can be used in a closed-loop PEMF system.

Smooth-muscle relaxation and changes in arterial compliance can alter segmental filling and pulse-wave morphology. A localized impedance signal may therefore change through:

- The pulsatile blood volume of the segment across the cardiac cycle, reflected in the amplitude parameters of the impedance pulse wave;
- The pulse arrival time (which also contains the cardiac pre-ejection period) and the maximum systolic upslope, reflected in its temporal parameters;
- The shape of the wave and its reflected (dicrotic) component, reflected in its morphological parameters—chiefly the dicrotic index and the timing of the dicrotic incisura and the inflection point (Section 3.5, Figure 4).

These relationships make impedance pulse features plausible candidate monitoring variables, but they do not make bioimpedance a direct assay of eNOS activity or capillary microcirculation. The mapping from molecular signalling to a specific impedance feature must be calibrated empirically against reference vascular measurements.

#### 3.3.4. Angiogenic and Remodeling Observations in Separate Models

Separate cellular and animal studies have reported changes in angiogenic markers or vascular-remodeling outcomes after particular electromagnetic exposures; these findings do not establish long-term vascular remodeling in patients receiving peripheral PEMF. Goto et al. showed that PEMF at 10 h of exposure per day for 7 days increases the levels of angiopoietin-2 (Ang-2) and fibroblast growth factor 2 (FGF-2) in mouse bone marrow, while VEGF and Ang-1 remain unchanged; such a marker profile is regarded by the authors as “physiological angiogenesis” without signs of hypoxic or inflammatory activation [62]. Vincenzi et al. described a HIF-1α-independent pathway of VEGF release in astrocytes upon stimulation at 75 Hz/1.5 mT [63], and Gerdesmeyer et al. (2022) showed that high-intensity electromagnetic transduction (EMTT, 80–150 mT) induces the production of VEGF and Ang-2 by human mesenchymal stromal cells [64]. Additionally, Cheng et al. described a specific mechanosensitive TRPV4-dependent pathway of endothelial protection against pyroptosis [50], and Wang et al. described a HIF-1α-dependent induction of CD31^hi^/Endomucin^hi^ endothelium [51].

Hyldahl et al. investigated human microglial responses to pulsed electric fields induced using transcranial pulsed electromagnetic fields (T-PEMF), reporting increased secretion of VEGF, IL-8, and GLP-1 and paracrine activation of endothelial cells [65]. These findings are included as mechanistic evidence from a cellular model and should not be interpreted as demonstrating improved limb perfusion or clinical benefit from peripheral PEMF treatment. In a separate PEMF study, Gessi et al. reported that in neuron-like PC12 cells under hypoxia, PEMF activates the p38/HSP70/CREB/BDNF/Bcl-2 pathway, promoting neuronal survival through a neuroprotective mechanism parallel to NO [66]. Park et al. (2022) showed activation of the ERK/CREB pathway upon induction of neurodifferentiation of mesenchymal stem cells in cerebral ischemia models [67]. Vincenzi et al. (2017) systematized the data on the action of low-frequency, low-energy PEMF on neurons and microglia: under hypoxia and inflammation, PEMF reduces the levels of HIF-1α, ROS, TNF-α, and IL-1β [68].

The work of Sayin et al. (2026), in a model of oral mucosal wounds in Wistar rats (*n* = 32), showed that PEMF induces a complex activation of the growth factors VEGF, EGF, FGF, and PDGF, promoting accelerated healing [69]. Li et al. (2020), in a novel PEMF regimen in distraction osteogenesis, showed activation of CD31-positive vessels and VEGF in the regenerate zone [70]. Taken together, these observations indicate that PEMF can engage several angiogenic mediators (VEGF, FGF, Ang-2, and BDNF) in individual models; however, because the studies span different models, tissues, species, and exposure conditions, they do not establish a single common PEMF angiogenic program, and the pathway map in Figure 3 should be read as a set of separately observed links rather than a validated cascade.

#### 3.3.5. Contextual Anti-Inflammatory Pathways

Adenosine-receptor and other anti-inflammatory pathways have been reported in chondrocyte, neural, and preclinical models [71,72]. These data may inform biological hypotheses, but they do not demonstrate that inflammation-mediated changes in peripheral circulation are captured by a specific limb bioimpedance feature.

Taken together, the mechanistic literature supports a testable hypothesis: an exposure-induced vascular response may be reflected in segmental impedance and pulse morphology. It does not establish that a bioimpedance feature is a specific surrogate for eNOS activity, nor that acute pulsatile and long-term remodeling effects can be separated without multimodal validation.

### 3.4. Clinical Populations and Nosological Targets

The intervention corpus of 32 reports (30 study/report clusters) was clinically concentrated in PAD and diabetes-related distal circulatory impairment, with smaller proxy, animal, and technical evidence groups; second-pass additions extended it to chemotherapy-induced polyneuropathy, lymphedema, post-thrombotic syndrome, and critical-illness microcirculation. The IEEE additions concern ex vivo rabbit RBCs in microchannels and add technical evidence rather than clinical studies.

#### 3.4.1. Direct Clinical Evidence

Fifteen studies evaluated direct peripheral-circulation outcomes in participants with PAD, diabetic foot ulcer, diabetic neuropathy, chemotherapy-induced polyneuropathy, lower-limb lymphedema, post-thrombotic syndrome, or healthy-volunteer acute models [22,23,24,25,26,27,28,29,30,31,32,33,34,35,36]. The strongest endpoints were instrumental—LDF, PPG, ABI/TBI, skin perfusion pressure, and small-vessel imaging—rather than symptoms alone.

#### 3.4.2. Peripheral Arterial Disease and Claudication

Small clinical studies in Fontaine stage II PAD reported improved ABI and walking performance with PEMF used alongside drug therapy or exercise-related care [26,27,29]. The direction is encouraging, but sham control, allocation concealment, blinding, independent replication, and separation from co-interventions remain insufficient. A further randomized complex-physical-therapy study added at the second pass reported improved claudication distance without a significant ankle-brachial-index change [36].

#### 3.4.3. Diabetes-Related Distal Circulation

Diabetic-foot and neuropathy studies used LDF, PPG, capillary imaging, ABI/TBI, and skin perfusion pressure [22,23,24,25,28,30]. Findings ranged from positive to mixed or null, including a non-significant vascular endpoint in the largest sham-controlled trial [25]. The second-pass additions in chemotherapy-induced polyneuropathy, pediatric diabetic polyneuropathy, lymphedema, and post-thrombotic syndrome reported positive LDF-derived microcirculatory outcomes [32,33,34,35], with the usual caveats of single-center design, co-interventions, and incomplete physical dosimetry. This range of results should be retained rather than collapsed into a single efficacy percentage.

#### 3.4.4. Endothelial and Cardiovascular Proxies

Six clinical support reports (five studies) measured flow-mediated dilation, plasma NO, blood pressure, arterial stiffness, temperature, sublingual microvascular flow, skin perfusion during stimulation, venous flow, or related proxies [4,18,19,37,38,39]. Two of these reports appear to describe the same trial and are conservatively counted as one study [18,19]. These outcomes can inform vascular function but are not equivalent to direct limb perfusion; they were therefore treated as a distinct evidence role.

#### 3.4.5. Preclinical and Veterinary Evidence

Three rodent hindlimb-ischemia studies, one rabbit carotid-sinus stimulation study with an intensity-dependent blood-pressure response [42], and one small equine sham-controlled study evaluated perfusion-related temperature ratios, capillary density, angiogenic signalling, blood pressure, or distal vessel diameter [2,40,41,42,43]. They support biological responsiveness while remaining limited by acute surgical models, indirect thermographic outcomes, and species differences.

#### 3.4.6. Technical and Pilot Evidence

Six technical/pilot reports, grouped into five study/report clusters, were included. Four used acute high-field stimulation in a stenosed microfluidic channel or in human finger/hand pulse-wave experiments, or characterized stimulation-related artifacts in peripheral hemodynamic signals [44,45,46,47]; their outcomes were mixed and sometimes transient. Two related microchannel reports quantified red-blood-cell velocity and sedimentation in rabbit blood under 1 Hz pulsed magnetic exposure and described higher velocities after exposure [20,21]. The value of this group is technical—showing parameter-sensitive hemodynamic, waveform, or flow responses—not therapeutic efficacy, and in vitro observations do not establish in vivo perfusion effects.

#### 3.4.7. Outcome-Measure Heterogeneity

The corpus spans macrovascular pressure indices, microvascular perfusion, vessel calibre, pulse morphology, temperature, and functional walking outcomes. These measures operate at different physiological scales. LDF and capillary imaging address local perfusion; ABI/TBI address pressure ratios; PPG and bioimpedance address optical or electrical pulse-related signals; temperature is an indirect proxy. Their results should not be pooled without a pre-specified causal and measurement model.

#### 3.4.8. Evidence Limitations

Clinical samples were generally small, sham control was uncommon, co-interventions were frequent, and six reports were charted from abstracts only. Table 4 summarizes the quality characteristics of the corpus by evidence category; per-report details, including randomization, control condition, and blinding as reported, are given in Table S3. Physical dosimetry was incomplete in several reports. These features make the evidence suitable for hypothesis generation and trial design but insufficient for a definitive clinical protocol.

**Table 4:** Reported design characteristics of the core intervention corpus by evidence category. Randomization, control condition, blinding and sample size are summarized per study/report cluster using the stated linkage conventions. The two linked clinical-trial reports are counted once, and the Jo pair is conservatively grouped without establishing sample identity. The final column counts reports. Both complete Jo publications were available, although the conference report is abbreviated. These characteristics are not a formal risk-of-bias assessment. Per-report details are provided in Table S3. NR, not reported.

Evidence Category	Reports (Study/Report Clusters)	Randomized	Sham/Placebo-Controlled	Blinding Reported	Sample Sizes	Full-Text Basis/Abstract-Only
Direct clinical	15 (15)	10	4	3	Median 47 (13–182)	12/3
Clinical support or proxy	6 (5)	1	2	2	Median 25 (10–55)	5/1
Preclinical/veterinary	5 (5)	3	2	0	16–42 animals; one NR	4/1
Technical/pilot	6 (5)	0	0	0	One *n* = 20; others bench or NR	5/1
Total	32 (30)	14	8	5	—	26/6

Overall, PAD and diabetes-related distal circulatory impairment are the most relevant populations for further study. Future trials should use objective peripheral-circulation endpoints, pre-specified dose comparisons, sham control, and transparent reporting of co-interventions and field dosimetry.

### 3.5. Bioimpedance and Impedance Plethysmography for Peripheral-Circulation Monitoring

A targeted technical mapping of bioimpedance records was conducted separately from the intervention corpus. Selected full-text systems and methodological reviews define measurement options and limitations; they are not evidence that bioimpedance improves outcomes or that it has already been validated during PEMF exposure.

#### 3.5.1. Multichannel Bioimpedance Systems

Hammoud et al. described the REO-32 multichannel bioimpedance system for simultaneous recording from multiple body segments [5]. The platform uses tetrapolar measurement at 100 kHz and extracts characteristic fiducial points and indices from cardiac-synchronous impedance waveforms. Within this review, it is treated as one technical example, not as a privileged reference standard.

- DKI = AI/AC—the dicrotic index, proposed in this device’s interpretive framework as an indicator of small-vessel arteriolar tone;
- DCI = AD/AC—the diastolic index, proposed as an indicator of venous outflow.

The platform classifies impedance pulse morphology using DKI and DCI and also records ECG and transthoracic impedance to support cycle and respiratory alignment [5]. These indices are device-specific signal features; their interpretation as small-vessel tone or venous outflow requires external validation against reference vascular methods.

The work of Langer et al. describes a parallel multichannel system, MBM (Multichannel Bioimpedance Monitor), recording bioimpedance at 16 body points simultaneously using frequency multiplexing (49, 50, 51 kHz) [6]. The authors complement bioimpedance with continuous blood-pressure measurement (the Peñáz method), 12-lead ECG, and phonocardiography. The extracted parameters include Z_0_ (a slow change reflecting blood volume) and −dZ/dt_max (a fast change, an analog of flow), as well as pulse-wave velocity (PWV) from the time interval between −dZ/dt peaks at two points. A key observation: deep controlled breathing (0.1 Hz/5 s per phase) induces pronounced synchronization of blood-volume distribution between the limbs, and PWV changes differently in the abdominal aorta and in the distal limb segments, confirming the anatomical heterogeneity of the vasomotor response. This is methodologically important for adaptive systems: the local bioimpedance signal in the treated limb cannot be fully predicted from the systemic hemodynamic state, which justifies the need for local (rather than central) recording.

#### 3.5.2. Vector Bioimpedance and Wearable Devices

Hersek, Töreyin, and Inan described a wearable vector-bioimpedance system for the knee with separate resistance and reactance recording at 50 kHz [7]. The system captured slow fluid-related changes and a cardiac-synchronous component; a cold-pressor experiment altered pulsatile resistance, demonstrating physiological sensitivity in a small sample. The study supports measurement feasibility but does not establish accuracy for microcirculation, and its sample size was insufficient for clinical performance claims.

Kalvøy, Hisdal, and Tronstad described tetrapolar impedance plethysmography at the toe using flexible printed electrodes and a high-sampling-rate analyzer [8]. The work demonstrates feasibility at a clinically relevant distal site. It does not by itself validate the signal as a measure of capillary perfusion or as a feedback variable during electromagnetic exposure.

#### 3.5.3. Impedance Cardiography in Pharmacological Trials

Yamaleyeva et al. used thoracic impedance cardiography alongside tonometry, venous-occlusion plethysmography, and LDF during a vasoactive infusion [73]. The study is a useful multimodal-validation precedent, but thoracic ICG is a systemic hemodynamic method and should not be treated as direct validation of localized limb bioimpedance.

#### 3.5.4. Consolidating Methodological Recommendations

The review of Anand, Yu, Lowe, and Kalra (2021) systematizes methods of bioimpedance hemodynamic monitoring, including ICG, segmental BIA, and localized BIA [9]. The authors highlight three key recommendations: (i) an operating frequency range of 1 kHz–1 MHz, with a preference for multifrequency analysis; (ii) a tetrapolar configuration as the universal standard; and (iii) localized limb bioimpedance as having methodological advantages over thoracic ICG owing to reduced reliance on geometric assumptions. Multifrequency analysis may help to separate the contributions of blood volume, erythrocyte orientation, flow velocity, and temperature to blood conductivity, but only through an appropriate model and experimental validation; it does not separate them automatically. The principal limitations noted are geometric assumptions, calibration inaccuracies, and temperature dependence. The review’s key conclusion is that for localized limb application, multifrequency bioimpedance is methodologically preferable.

#### 3.5.5. The Pulse-Wave Parameter Set as a Candidate Feedback Signal

Across the technical literature, candidate features can be grouped into amplitude, temporal, and morphological families (Figure 4 and Table 5). Examples include normalized pulse amplitude (ΔZ/Z_0_), ECG-referenced pulse arrival time, time-to-peak, relative fiducial timing, dicrotic index, area ratios, and symmetry measures. This is a candidate feature set, not a validated twelve-variable controller.

Absolute amplitudes depend strongly on segment geometry, baseline impedance, electrode placement, contact, temperature, and fluid distribution. Temporal and ratiometric features may be less sensitive to multiplicative drift, but their expected direction under vasoconstriction or vasodilation cannot be assumed to transfer unchanged from pressure or optical pulse waves to impedance waveforms.

For adaptive control, the most attractive features are those computable cycle by cycle and robust to baseline drift. Candidate metrics include normalized amplitude, relative incisura timing, pulse arrival time, and morphology ratios. Selection should be data-driven and pre-specified after repeatability and responsiveness studies; no feature can currently be designated a direct terminal readout of a PEMF–NO pathway.

The principal metrological gap is the absence of established within-session and between-session repeatability, intraclass correlation, minimal detectable change, and reference-method agreement for the proposed limb impedance features under PEMF exposure. A signal cannot serve as a reliable control variable until its measurement error and clinically meaningful change are quantified.

Three qualifications constrain the practical use of this set as a control signal. First, the twelve parameters are not statistically independent: within the amplitude family, the pulse amplitude, the area, and the mean ΔZ are strongly collinear (the mean is by definition the area divided by cycle duration, and the area is dominated by the amplitude), and the maximum systolic upslope scales with the amplitude as well; a working feedback signal would therefore be reduced to an orthogonal subset rather than track all twelve. Second, the absolute amplitude parameters carry physical units (Ω or mΩ for amplitudes, Ω·s for pulse areas, and Ω/s for the maximum slope), but their values depend on the baseline segmental impedance Z_0_, electrode placement, and contact conditions, and they drift between and within sessions; the ratiometric and temporal parameters (the dicrotic and area indices, the pulse arrival time, and the relative timing of the inflection point and incisura) may reduce sensitivity to selected scaling effects, but improved repeatability has not been established for the proposed use during PEMF exposure. Any amplitude normalization (for example, as ΔZ/Z_0_) must be evaluated with the same repeatability and responsiveness tests as the other candidate features. Third, a point of nomenclature requires care. The ratio of incisura amplitude to peak amplitude, used throughout this review and denoted here as the dicrotic index (DI), is not the augmentation index (AIx) of the classical pressure-pulse literature, which is the ratio of augmentation (second systolic) pressure to pulse pressure [12,13]. The two are sometimes conflated. Because the impedance waveform is neither a pressure nor a volume pulse, the transfer of either index onto it is an assumption that requires empirical validation before the metric is used to drive field parameters; the present review adopts DI and reserves AIx for its original definition.

**Table 5:** Candidate impedance pulse-wave features defined in Figure 4, grouped by family, with their first-order sensitivity to the multiplicative and additive drift discussed in Section 3.5.5. Absolute amplitudes are expressed in Ω (or mΩ), pulse areas in Ω·s, and the maximum slope in Ω/s; these values depend on Z_0_, electrode placement, and contact; ratiometric and timing features are self-normalizing to first order.

Code	Family	Feature	Definition	Drift Sensitivity
A1	Amplitude	Pulse amplitude	Peak ΔZ within the cycle (foot to systolic peak)	Absolute; requires normalization (ΔZ/Z_0_)
A2	Amplitude	Pulse area	∫ΔZ dt over the cycle (S1 + S2)	Absolute
A3	Amplitude	Mean ΔZ	Area divided by cycle duration	Absolute; collinear with A1 and A2
B4	Temporal	ECG-referenced pulse arrival time (PAT)	Delay from the ECG R-wave to the pulse foot; includes the cardiac pre-ejection period	Timing; gain-independent
B5	Temporal	Time-to-peak	Foot to systolic peak	Timing; gain-independent
B6	Temporal	Maximum upslope	Maximum dZ/dt on the upstroke	Absolute; scales with A1
B7	Temporal	Inflection time	Time of the inflection point on the downstroke	Timing; gain-independent
C8	Morphological	Dicrotic index (DI)	Incisura amplitude/A1	Ratiometric
C9	Morphological	Area index (IPA)	S2/S1	Ratiometric
C10	Morphological	Waveform symmetry	Upstroke versus downstroke shape (e.g., rise/decay time ratio)	Ratiometric
C11	Morphological	Incisura amplitude	ΔZ at the dicrotic incisura	Absolute
C12	Morphological	Incisura time	Position of the incisura within the cycle	Timing; gain-independent

#### 3.5.6. Convergence of Technical Parameters

Across selected technical exemplars, three recurrent design choices were identified:

- Probing frequency: approximately 50–100 kHz in several single-frequency systems [5,6,7,8], with multifrequency measurement recommended when separation of tissue contributions is required [9];
- Electrode configuration: tetrapolar measurement is preferred for reducing the influence of electrode contact impedance, although placement and geometry remain important sources of variation;
- Probing current and sampling: sub-mA to approximately 1 mA currents and sampling rates sufficient to preserve cardiac morphology are common, but compliance and safety must be assessed for the complete device and intended use.

**Figure 5:**
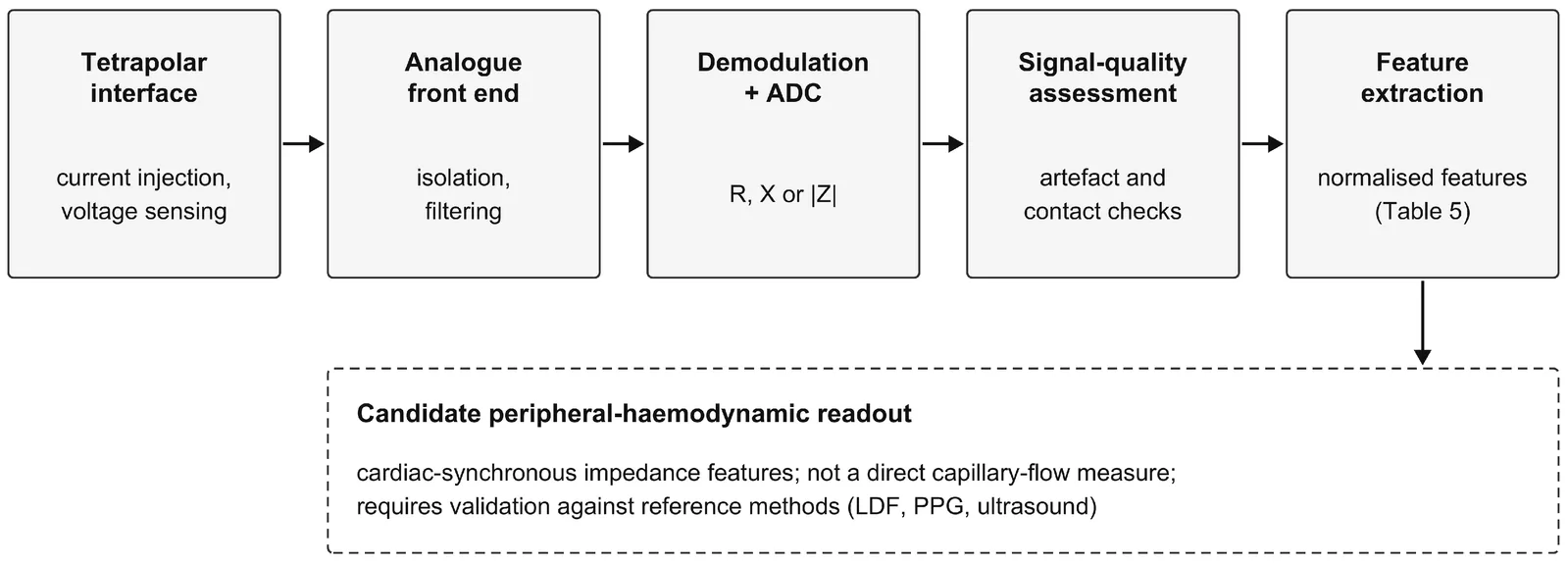
Representative tetrapolar bioimpedance signal chain for segmental peripheral-hemodynamic monitoring. Existing systems can extract impedance and pulse-related features, but the architecture shown is a technical template rather than a validated closed-loop medical device.

In the selected technical literature, bioimpedance was used as a diagnostic or monitoring signal rather than to adapt a therapeutic PEMF exposure in real time. Figure 5 shows a representative signal chain for a localized system of this type, and Table 6 summarizes recurring engineering choices and the cautions that accompany them.

### 3.6. Impedance Plethysmography and Rheovasography

A targeted set of impedance plethysmography and rheovasography studies was examined to characterize the pulsatile measurement modality. These records were treated as technical context rather than intervention-effect studies.

The work of Manasyan et al., which assessed endothelial function by peripheral arterial tonometry, also used polyhepatography for recording intrahepatic microcirculation [74]. The work of Nalobina et al. concerns rheovasography in young athletes with infantile cerebral palsy (*n* = 15, swimmers and alpine skiers) on the RGPA-6/12 platform [75]. Extracted parameters: volumetric blood flow (Qv), maximal and slow filling velocities (Vmax, Vs), baseline resistance R_0_, and characteristics of venous outflow. Rheovasography revealed a sport-specific adaptation of tone: alpine skiers showed increased tone and baseline resistance of the lower limbs, while swimmers showed increased Qv of the shoulder girdle with concurrent impairment of leg venous outflow. The work is important as a methodological example: RVG was able to distinguish local adaptations of tone within a single subject in a comparatively small sample.

The work of Małecki et al. describes a multimodal study of patients with essential thrombocythemia (*n* = 45 + *n* = 30 controls) using impedance plethysmography, photoplethysmography, applanation tonometry, and infrared thermography of the upper limbs [76]. The Dauer classification of pulse-wave types was applied (A = normal, B = impact, C = pointed). A reduction in skin temperature (31.04 vs. 32.45 °C), an increase in the temperature gradient (1.82 vs. 0.11 °C), and a reduction in the amplitude of the volumetric PPG pulse (0.25 vs. 0.74%) were recorded; the type-B pulse correlates with the temperature gradient. The concordance of IPG, PPG, and thermography is consistent with the bioimpedance signal reflecting real changes in peripheral blood flow, although agreement between modalities does not by itself establish the accuracy of impedance in tracking flow changes.

The work of Dolganova et al. concerns tetrapolar rheovasography of the lower limbs in patients with femoral pseudarthrosis under combined osteosynthesis (*n* = 26) [77]. Extracted indices: volumetric blood flow of the thigh and lower leg, pulsatility index PI = 12 ± 3.5, resistance index RI = 0.9 ± 0.1, and blood-flow type (main/collateral). Rheovasography distinguished local hyperemia in the regenerate zone from systemic hemodynamics that showed no substantial change, suggesting a useful methodological property: the potential to separate the local vascular response in the intervention zone from the general systemic background. This property is fundamental for tasks of adaptive PEMF control, where precise tracking of the local vascular response in the treated limb is required.

Together, these studies support the feasibility of detecting local and pulse-related hemodynamic variation with impedance methods. They do not establish direct measurement of capillary microcirculation, nor do they provide the repeatability, responsiveness, and artifact performance required for closed-loop use during PEMF.

### 3.7. Closed Loops and Adaptive Control in Electromagnetic Therapy

Adaptive-control records were mapped as a contextual evidence stream. They included manual PEMF individualization, cardiac-synchronized impedance-guided exposure, adaptive TMS, and sensor-rich engineering platforms. None was counted as evidence of PEMF efficacy for peripheral circulation.

#### 3.7.1. Cardiac-Cycle-Synchronized, Rheovasography-Guided PEMF

A 2009 antecedent described pulsed electromagnetic exposure synchronized to the cardiac cycle, with rheovasography used to select an individual phase before treatment [16]. The report is historically relevant because it combines PEMF timing with an impedance-derived vascular signal. It lies outside the review window, and independent replication or real-time adaptation of field parameters was not identified.

This antecedent is best classified as individualized open-loop control: measurement informs a pre-session timing choice, but the controller does not continuously update frequency, field strength, or waveform from an evolving limb impedance feature. It therefore motivates, but does not validate, the adaptive system proposed here.

#### 3.7.2. Manual Magneto-PAT Biofeedback

Machnia, Cichoń, and Miller reported a single clinical case using manual adjustment of an ONDAMED^®^ PEMF device according to a qualitative radial-pulse response [78]. The report demonstrates individualized parameter selection from a peripheral signal, but the signal was subjectively interpreted, adaptation was not continuous, and a single case cannot establish efficacy.

#### 3.7.3. Precision TMS with Artificial Intelligence: An Architectural Template

Liu, Hu, and Bao reviewed adaptive TMS architectures that combine measurement, signal analysis, and adaptation [79]. The general control structure is transferable, but neuroimaging or EEG feedback for a central target does not validate limb bioimpedance feedback or peripheral-circulation outcomes.

#### 3.7.4. Open-Source Technical Implementations

Huang et al. described an open, sensor-rich platform for adaptive electromagnetic stimulation [80]. Its real-time software and hardware architecture is a useful engineering precedent, but the biological target and feedback signals differ from those required for peripheral circulation.

#### 3.7.5. Closed-Loop Control of an Applied Field Using a Measured Biological Response

Marquez et al. demonstrated closed-loop adjustment of an applied electric field from a measured cellular response in vitro [81]. This is a control-theoretic precedent rather than a PEMF or vascular study. It illustrates actuator constraints and adaptive control but cannot be used as evidence for clinical transfer.

The vocabulary mismatch across therapy, neuromodulation, bioimpedance, and control engineering limits recall. Consequently, the absence statement in this review is bounded by the databases and queries used, and should be retested in registries and patent sources, which remain unsearched. The supplementary IEEE Xplore search (Section 2.6) illustrates this limit: it retrieved two precedents that the main strategy had not captured (Section 3.7.6).

#### 3.7.6. On-Line Impedance Monitoring and Biotechnical Feedback in Magnetotherapy

Mishin et al. described four-lead impedance measurements before, during, and after intravaginal pulsed magnetic treatment, with amplitude and phase estimates stored every 4 s [82]. Preliminary patient recordings showed decreasing impedance, but the report did not specify cohort size, validate the inferred perfusion change against an independent vascular measure, or demonstrate automatic adjustment of the therapeutic field. This is an early clinical monitoring precedent outside the limb setting and the core review period. Concurrent recording during a treatment session should not be interpreted as validated artifact-free acquisition during each magnetic pulse.

Gurzhin et al. described pulse-oximeter-based cardiac monitoring and ultrasound respiratory-signal processing in the Multimag system and discussed bioadaptive regulation [83]. Their respiratory phase-detection algorithm was explicitly unsuitable for action synchronization because of its delay. The report did not demonstrate impedance-guided adaptation, specify the therapeutic field parameters, or provide a controlled evaluation of benefit from adaptive treatment. Both reports were assessed in full text. They establish relevant monitoring and proposed-feedback precedents, but neither demonstrates a validated limb-bioimpedance controller targeting peripheral perfusion.

#### 3.7.7. A Map of Existing Precedents

A summary of the six architectural lines described is given in Table 7.

The precedents cover complementary elements: impedance-guided phase selection [16], manual peripheral-pulse guidance [78], algorithmic adaptation in TMS [79], real-time sensor integration [80], on-line impedance monitoring during pulsed magnetic exposure [82], and proposed pulse/rhythm- and respiration-informed feedback for adapting magnetotherapy [83]. The cardiac-synchronized, rheovasography-guided method [16] and on-line impedance monitoring during pulsed magnetic therapy [82] are the closest impedance-related antecedents, but the former remains open-loop, and the latter monitored impedance without adapting the field. None used validated limb bioimpedance features to update PEMF parameters continuously for a peripheral-circulation target.

**Figure 6:**
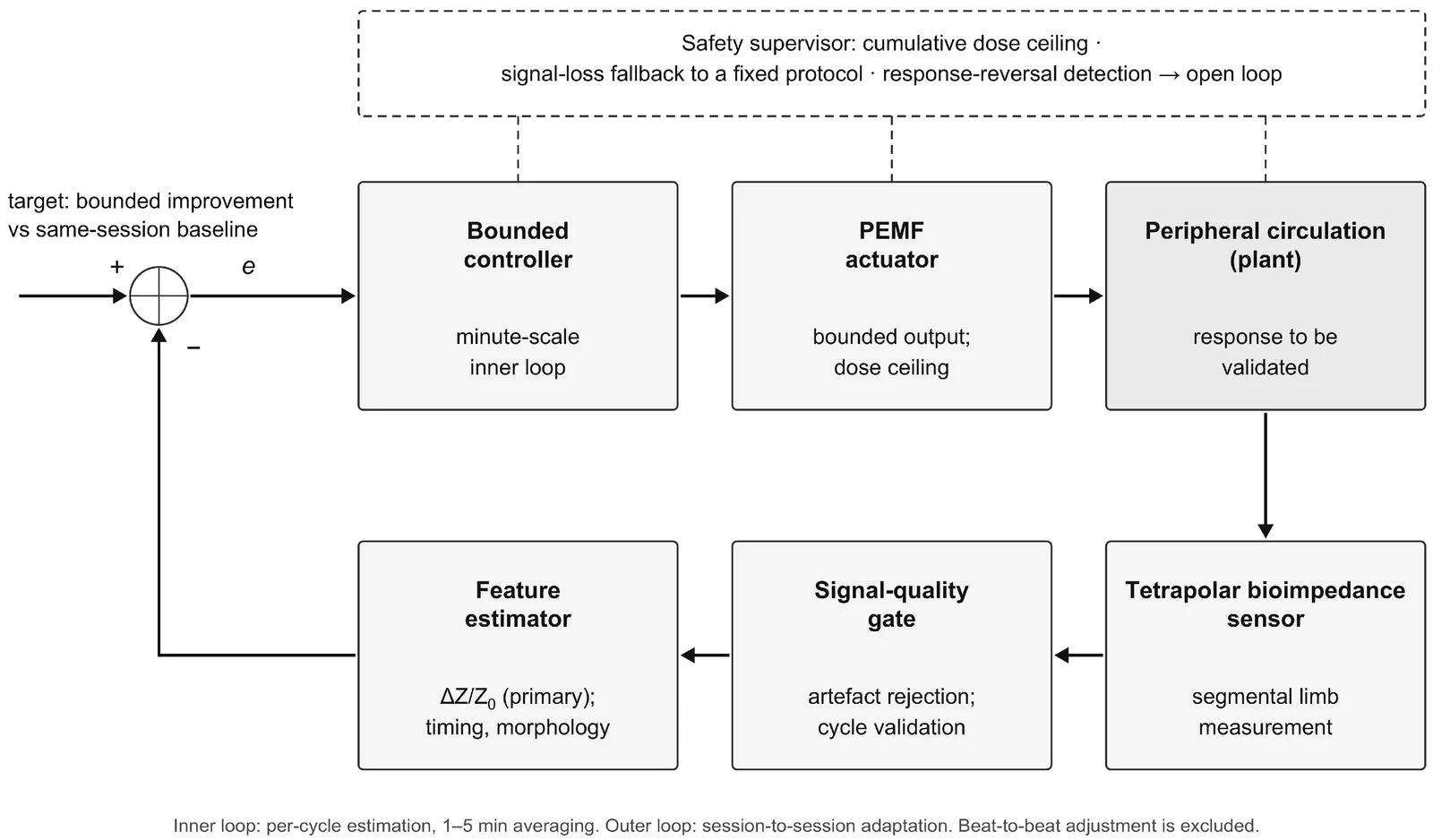
Hypothesis-generating adaptive PEMF–bioimpedance architecture. All controller, sensor, signal-quality, target, and safety-supervision blocks denote proposed functions requiring validation. Normalized amplitude ΔZ/Z_0_ is a candidate feedback feature, not a validated surrogate or established treatment target. The pre-exposure recording supplies a within-subject reference; the desired direction, target bounds, and averaging windows must be determined prospectively (Section 4.6). Invalid feedback must inhibit adaptive updates. Pausing exposure or entering a fixed mode requires a device-specific validated fault response. Exposure limits and response-reversal handling are engineering requirements, not established settings. Historical and contemporary precedents [16,78,79,80,82,83] support individual architectural elements but do not validate this integrated system for peripheral circulation.

The wider contextual literature is dominated by adaptive TMS, deep-brain stimulation, and electrical stimulation with neural or biomechanical feedback. Most contextual records added by the supplementary IEEE Xplore search belonged to the same categories, chiefly EEG-triggered or state-dependent TMS. These fields show that closed-loop stimulation is technically feasible, while also underscoring that each sensor–target–actuator combination requires its own validation. Figure 6 places the proposed adaptive architecture alongside these contextual precedents.

## 4. Discussion

### 4.1. Synthesis of the Core PEMF Evidence for Peripheral Circulation

The 32 core intervention reports provide a heterogeneous, early evidence base rather than a settled therapeutic protocol. The direction of reported circulation outcomes was heterogeneous across designs and settings (Section 3.1); these descriptive patterns span different designs and cannot be interpreted as a pooled success rate. Because no formal risk-of-bias assessment was performed and PEMF parameters varied widely across reports, the corpus does not support definitive conclusions about clinical efficacy. Clinical evidence is concentrated in PAD and diabetes-related distal circulatory impairment, with important limitations in sample size, sham control, co-interventions, and dosimetry.

Table 8 summarizes observed exposure patterns and their interpretation. It deliberately avoids a recommendation column: recurrence near 10–30 Hz and low-mT fields may guide prospective dose-finding, but the corpus does not establish a universal frequency, field strength, pulse shape, session duration, or course length.

### 4.2. Bioimpedance as a Technically Promising but Insufficiently Validated Instrument

Localized bioimpedance is technologically capable of recording segmental impedance and cardiac-synchronous waveforms, and tetrapolar systems around 50–100 kHz recur in the technical literature [5,6,7,8,9]. However, technological availability should not be confused with clinical validation. Bioimpedance does not directly quantify capillary microcirculation, and the proposed pulse features lack established repeatability, minimal detectable change, and responsiveness during PEMF exposure.

The technical obstacle is interference between a stimulating field and a sensitive impedance front end operating in the same tissue volume. Simultaneous stimulation and acquisition present a device-specific problem. Section 4.5 identifies coupling mechanisms and verification requirements; it does not establish artifact-free operation or a guaranteed signal-to-interference margin.

### 4.3. Biophysical Plausibility and the Unresolved PEMF–Bioimpedance Coupling

A plausible biophysical hypothesis links PEMF-sensitive endothelial or smooth-muscle pathways to changes in peripheral hemodynamics and then to pulse-related impedance features. This relationship is indirect and non-specific: impedance is also influenced by geometry, tissue fluid, temperature, erythrocyte orientation, and electrode contact. It should therefore be tested against LDF, PPG, pressure indices, or ultrasound rather than asserted as a direct consequence of a particular molecular cascade.

The unmeasured link is the simultaneous observation of limb PEMF exposure, a reference vascular response, and a localized impedance pulse response. Evidence exists for individual components in separate studies, but no core study closes the chain. The adaptive-control concept is therefore biologically plausible and empirically untested. To state the distinction explicitly: biological plausibility of the individual links does not constitute evidence of a validated feedback relationship, and the complete sequence PEMF → vascular response → hemodynamic change → impedance change → PEMF adjustment has not been demonstrated experimentally in any study identified by this review.

### 4.4. Existing Architectural Templates for Closed Loops

Closed-loop stimulation is not a new engineering problem, but the specific sensor–target–actuator combination reviewed here remains unvalidated. The cardiac-synchronized, rheovasography-guided method [16] provides the closest historical coupling of PEMF timing and impedance information, while other precedents contribute manual pulse guidance, algorithmic adaptation, or real-time sensing [78,79,80], on-line impedance monitoring during pulsed magnetic therapy [82], or proposed pulse/rhythm- and respiration-informed feedback for adapting magnetotherapy [83]. None demonstrates continuous adjustment of PEMF parameters from validated limb bioimpedance features for peripheral circulation.

A transferable architecture consists of measurement, signal-quality assessment, feature estimation, a bounded controller, and an actuator [79]. Transfer is not automatic: the candidate impedance features, artifact rejection, safety limits, setpoints, and fallback must be defined and prospectively validated for the limb and clinical population.

### 4.5. Engineering Requirements for Simultaneous Stimulation and Measurement

Simultaneous acquisition is a design objective that requires verification with the intended applicator, electrodes, leads, and front end. Under a uniform-field approximation, inductive pickup scales with effective loop area and the rate of change of magnetic flux density. An illustrative 1.5 mT change over 200 µs gives an average slew rate of approximately 7.5 T s−1; the peak rate depends on the actual pulse edges. Neither value alone determines the voltage reaching the amplifier or the residual artifact after demodulation. Those quantities depend on coupling geometry, electrode interfaces, analog bandwidth, gain, and recovery behavior.

Synchronous demodulation can reject interference outside its effective detection band while the acquisition chain remains linear. It does not by itself establish immunity to pulse harmonics near the carrier, common-mode conversion, aliasing, amplifier saturation, or prolonged recovery. Consequently, no quantitative rejection ratio or signal margin is assigned to the proposed system without a complete circuit model and bench measurements. Lead routing, reduced loop area, appropriate input protection and filtering, adequate dynamic range, and synchronized acquisition are candidate mitigations whose performance must be measured.

Verification should begin with stable electrical loads and tissue-equivalent phantoms exposed to the intended waveforms, with field-on and field-off recordings and direct inspection of clipping, recovery, and spurious feature changes. Testing should then address electrode coupling and movement under realistic conditions. If continuous acquisition cannot meet prespecified signal-quality criteria, pulse-edge blanking or temporally separated stimulation and measurement should be evaluated. The lost samples, recovery interval, and effects on pulse-feature estimation must be reported.

Human feasibility testing should combine impedance with an independently validated vascular reference and synchronized stimulation logs. Sham exposure, a reference site where appropriate, and recording of movement, temperature, respiration, and cardiovascular changes can help distinguish instrument artifacts from physiological responses. Genuine stimulation-related physiology is not necessarily a beneficial vascular response. Adaptive updates should remain disabled when measurement validity is uncertain.

The implantable-device interference and physical-twin studies [84,85] provide adjacent electromagnetic-compatibility context, not validation of the proposed limb impedance front end. A separate physiological caution comes from magnetic-stimulation-related hemodynamic measurements [47]: stimulation-associated local and systemic changes can occur even when a signal is electromagnetically clean. Instrument interference, nonspecific physiological responses, and a target vascular effect therefore require separate assessment.

### 4.6. Control-Theoretic Requirements and Unresolved Problems

Describing the proposed system as closed-loop control imposes obligations that the literature reviewed does not yet discharge. Four are fundamental.

The first is that the sign of the plant gain may depend on baseline state. The cardiac-synchronized method of Section 3.7.1 reported arterial dilation near Δt = 0 and a reversal toward constriction near Δt ≈ 0.6T, while the clinical literature includes vasodilatory and context-dependent responses. For control design, this raises the possibility that a fixed-polarity controller could be inappropriate across different baseline vascular states. A practical system would therefore require an identification phase, gain scheduling, or a supervisory rule that detects response reversal and reverts to open loop. None has been demonstrated for PEMF. Grote et al. [86] found that short-term autonomic responses after exercise depended on pre-exposure autonomic tone. A review of electromagnetic-field therapy in diabetes described potentially “normalising” vascular effects, but that interpretation drew substantially on static-field evidence and does not demonstrate a sign-changing PEMF plant [87]. These sources motivate baseline-state stratification; they do not establish the control law.

A second unresolved requirement is the definition and validation of the feedback variable and target. Normalized pulse amplitude, ΔZpulse/Zbase, is a candidate feature, where ΔZpulse denotes a consistently defined cardiac-synchronous amplitude, and Zbase is the contemporaneous baseline impedance of the measured segment. The selected impedance component, measurement frequency, and pulse-amplitude extraction rule must be specified consistently for the numerator and denominator. Normalization may reduce some multiplicative scaling effects, but it does not eliminate electrode-position, contact, geometry, temperature, fluid-shift, or additive artifact effects. Within-session and between-session error, responsiveness, and the stability of the denominator must therefore be established before controller use.

The pre-exposure recording provides a within-subject reference, not a therapeutic target or a validated safety boundary. Neither a universal increase in normalized amplitude nor a corridor defined by mean ± 1 SD is justified as a treatment objective by the reviewed evidence. The sign and magnitude of a desirable change must be established against an appropriate vascular reference and interpreted within the intended population. A detectable change beyond measurement error is not necessarily a clinically beneficial change. Target trajectories, averaging windows, exposure limits, and stopping rules remain parameters for prospective identification and validation, rather than established settings of the proposed architecture. Morphological features may assist signal assessment only after their own performance has been characterized.

The third is the separation of time scales. The impedance pulse-wave parameters update once per cardiac cycle, of the order of one second; the acute vasomotor response to PEMF develops over minutes; the cumulative clinical effect requires weeks (Section 3.2.3). A controller that adjusts field parameters at the cycle rate would act largely on beat-to-beat variability rather than on the therapeutic process, and would be expected to inject noise into the exposure. A candidate architecture is hierarchical: per-cycle estimation, slower averaging for the inner loop, and session-to-session adaptation for the outer loop. These are engineering assumptions rather than validated update intervals. Rapid parameter adjustment at the beat-to-beat scale would therefore reflect physiological variability rather than therapeutic response and is explicitly excluded from the proposed architecture. The appropriate averaging windows are an empirical question that the data reviewed here cannot answer.

The fourth requirement is behavior under fault. Electrode detachment, movement, arrhythmia, and corrupted fiducial detection can invalidate feedback. Signal-quality rejection, output constraints, and a defined fault response require prospective verification. Invalid feedback must inhibit adaptive updates. Whether the device pauses exposure or enters a predefined fixed mode must be determined and validated through device-specific risk assessment; an untested fixed protocol is not assumed to be a safe fallback. Detecting response reversal is similarly a proposed supervisory function, not an established clinical control law.

The evidence-supported elements are the availability of localized impedance measurements and separately reported vascular responses to some PEMF regimens. The responsive feature, its directional relationship to a vascular endpoint, controller structure, and feedback update times are hypotheses. Numerical targets, exposure limits, gate thresholds, dose metrics, and fault-handling settings remain to be determined. No integrated device performance or clinical benefit is established by combining these elements in a block diagram.

### 4.7. Confounders in the Impedance Signal

Three properties of the measurement complicate its use as a control variable and are not adequately treated in the source literature.

The pulsatile impedance change is not purely volumetric. Alongside the change in blood volume, ΔZ carries contributions from the orientation and deformation of erythrocytes under shear and from flow velocity itself, so that the classical proportionality between pulse amplitude and segmental filling volume [15] is an approximation. Since PEMF is proposed to act on flow, a parameter that responds to both volume and velocity conflates the quantity of interest with a covariate of the intervention.

Temperature is a therapy-related confounder. Tissue resistivity varies with temperature, and applicator contact or prolonged exposure may produce local warming. Temperature should therefore be measured or compensated, and ratiometric or temporal features should be evaluated for robustness to slow drift rather than assumed immune to it.

Baseline drift over a session arises additionally from postural fluid shifts, hydration, and electrode polarization. The preference for self-normalizing parameters expressed in Section 3.5.5 addresses this in principle, but no study in the corpus demonstrates their stability over an hour-long recording under exposure. This is the same evidential gap identified for repeatability, and it should be closed by measurement rather than argument.

### 4.8. Safety, Contraindications, and Regulatory Considerations

Nominal probing current alone does not establish electrical safety. Frequency-dependent patient-current limits, isolation, leakage, electrode area, fault conditions, and the complete intended-use configuration must be verified under the applicable medical-electrical standards. The recurrent sub-mA to approximately 1 mA technical range is descriptive, not a declaration of compliance.

Therapeutic field exposure and measurement electronics require separate risk analyses. Patient treatment, operator and bystander exposure, implanted devices, field confinement, thermal effects, and electromagnetic compatibility must be evaluated for the intended device and use environment; literature recurrence of a field strength does not constitute a safety limit.

Contraindications cannot be derived from this scoping corpus. Any clinical system must follow its device-specific instructions and a formal clinical risk assessment, with particular attention to implanted electronic or ferromagnetic devices, pregnancy, neurologic vulnerability, malignancy, and unsupervised home use.

Closing the loop also changes the software and risk-control requirements. Regulatory classification depends on the intended purpose, degree of autonomy, exposure risk, and jurisdiction. Development should include traceable software lifecycle processes, human oversight, bounded outputs, fault detection, and a validated fallback protocol.

### 4.9. Staged Validation of the Proposed Architecture

The proposed architecture is hypothesis-generating. Advancement to adaptive treatment should depend on meeting prospectively specified criteria at successive stages.

First, establish within-session and between-session reliability of a small prespecified set of limb-impedance features without PEMF. Repeated measurements should address electrode replacement, posture, contact, temperature, and operator variation, and quantify absolute measurement error as well as relative reliability. Detectable-change thresholds should be derived for the intended measurement protocol rather than borrowed from unrelated signals.

Second, assess responsiveness and vascular meaning against a reference method selected for the target endpoint, such as LDF, PPG, or ultrasound. These methods measure different quantities and are not interchangeable. The analysis should distinguish association, calibrated agreement where applicable, and responsiveness to a defined vascular challenge; correlation alone does not establish that an impedance feature is a perfusion surrogate.

Third, characterize PEMF-related acquisition artifacts in bench and phantom tests before combined human exposure. Validate saturation recovery, electrode coupling, timing, filtering, signal-quality rejection, and any blanking or interleaving strategy. Subsequent feasibility recordings should demonstrate that apparent vascular-feature changes cannot be explained by the measurement artifact alone.

Fourth, investigate fixed PEMF regimens under sham-controlled conditions in a defined population. Prespecified dose comparisons should establish whether an independently measured vascular response occurs, its direction and time course, and whether the candidate impedance feature tracks it. If no reliable response or usable sensor relationship is demonstrated, progression to adaptive control is not justified.

Fifth, compare fixed and adaptive PEMF in a preregistered controlled trial only after the measurement and fixed-regimen stages are satisfactory. Before adaptive clinical testing, the implemented controller must undergo simulation and hardware-in-the-loop testing of its dynamic behavior, latency tolerance, exposure constraints, and failure handling, using prospectively specified acceptance criteria. The algorithm, target definition, permitted exposure range, cumulative-exposure constraints, signal-loss handling, and clinical endpoints should be fixed in advance. Reference vascular outcomes and patient-relevant outcomes should determine benefit; reaching the controller’s own impedance target is not sufficient evidence of therapeutic success.

### 4.10. Limitations of the Present Review

Two limitations are structural and apply to every stage of the review. First, no prospective protocol was registered. This limits the ability to distinguish decisions specified before screening from amendments made after the literature and reviewer feedback were examined; in particular, the multistream eligibility definitions and the revision-stage second-pass procedures create scope for post hoc classification changes. The decision logs improve traceability but do not remove that risk, and PRISMA-ScR reporting should not be interpreted as evidence of prospective protocol registration. Second, the main-search screening and charting used sequential investigator review with AI assistance rather than independent duplicate assessment, which may introduce selection and classification bias; independent human duplicate assessment was limited to the supplementary IEEE records (Section 2.6), and the IEEE agreement statistic does not measure screening reliability or recall in the main corpus.

Third, the main search covered Scopus, PubMed, and Web of Science; IEEE Xplore was added during revision as a supplementary search, but patent databases, trial registries, and grey literature were not searched. This is material for an absence claim in an engineering-facing topic: the IEEE Xplore search retrieved two relevant precedents that the main strategy had missed (Section 3.7.6), and comparable gaps may exist in the unsearched sources. The conclusion should therefore be read as “no study was identified by the present strategy,” not proof that no such system exists.

Fourth, retrieval was vocabulary-sensitive. The narrow Q6a formulation returned no records, while the broader Q6b retrieved many contextual neuromodulation and engineering studies. Known-item and citation-chaining tests should be expanded in future updates.

Fifth, targeted contextual searching—including Russian-language antecedents and technical papers—was not fully equivalent to the reproducible database query stream. These sources were therefore labeled as context and were not counted in the intervention corpus.

Sixth, six core reports were assessed from abstracts, limiting parameter and bias assessment. The full-text-only analysis narrows the evidence coverage but does not remove selective availability or design-related bias. Seventh, no formal risk-of-bias appraisal was applied. Eighth, the 2010–2026 scope excludes older intervention evidence and includes an incomplete final year; the no-date IEEE check cannot establish complete historical coverage in biomedical databases. Ninth, the extent of overlap between the Jo conference and journal reports is unknown. Conservative grouping and a separate-report sensitivity count make the counting assumption explicit but cannot resolve sample independence. Full publication access, including a complete one-page conference paper, does not establish methodological adequacy.

Finally, no quantitative synthesis was attempted. The descriptive direction counts give equal weight to unlike designs and may be affected by publication bias, selective outcome reporting, possible cohort overlap, and post hoc mechanistic interpretation.

### 4.11. Prospective Directions

On the basis of the systematization of the current data, several prospective directions for further research may be outlined:

The five stages in Section 4.9 define the proposed validation sequence, with controller verification before adaptive clinical testing. Screening consistency, metrological validation, and artifact characterization remain prerequisites. Subsequent research directions include:

The Jo microchannel reports [20,21] add quantitative ex vivo flow observations to the technical evidence, but their unreported biological sample sizes, uncertain overlap and absence of inferential statistics illustrate why additional reports do not establish clinical benefit. Prospective studies require explicit experimental units, replication, controls and independent vascular validation.

- Prospective validation of the relationship between PEMF exposure and normalized bioimpedance pulse features, with simultaneous LDF, PPG, pressure-index, or ultrasound measurements and explicit artifact testing;
- Development of transparent real-time algorithms for signal-quality assessment and bounded feature-based adaptation, with pre-specified setpoints, safety constraints, and fallback behavior;
- Comparative trials of fixed versus adaptive PEMF protocols in well-defined PAD or diabetes-related populations, after feasibility and measurement validity have been established;
- Evaluation of multifrequency localized bioimpedance where it adds identifiable information beyond a robust single-frequency pulse signal, with attention to temperature, geometry, and erythrocyte-orientation effects;
- Target-specific dose-finding studies that vary one exposure dimension at a time and distinguish acute hemodynamic responses from longer-term endothelial or structural adaptation;
- Subject-specific electromagnetic dosimetry of the limb, including field distribution, induced electric field, dB/dt, coil geometry, and tissue state;
- Standardized reporting and multimodal validation protocols, together with external deposition of search strings, screening decisions, and the complete study-charting table.

## 5. Conclusions

The main search identified 2979 records, retained 1750 after deduplication, and contributed 30 core reports representing 29 established studies. The supplementary IEEE search added two technical/in vitro reports and identified two architectural precedents. The combined corpus contains 32 reports, conservatively counted as 30 study/report clusters because independence of the Jo report pair is unconfirmed; the alternative counting convention is shown in Table S6.

Clinical studies reported heterogeneous peripheral-circulation or vascular-proxy findings, with positive, mixed, and null results across different designs and endpoints; they do not establish clinical efficacy. Preclinical and technical studies provide separate evidence of biological responsiveness or measurement feasibility and should not be treated as additional evidence of patient benefit. Low-frequency, low-mT regimens around 10–30 Hz recur, particularly near 15 Hz in small PAD and diabetes studies, but the literature does not establish a universal optimum for frequency, field strength, waveform, session duration, or course length.

Localized bioimpedance can record segmental impedance and cardiac-synchronous pulse features, but it does not directly measure capillary microcirculation. Candidate amplitude, temporal, and morphological features require repeatability, responsiveness, artefact, and reference-method validation before they can serve as control variables.

No study identified by the present strategy used validated limb bioimpedance pulse-wave features to adapt PEMF parameters continuously for a peripheral-circulation target. Historical impedance-guided and impedance-monitoring precedents, proposed feedback-adapted magnetotherapy, and contemporary adaptive-stimulation systems provide components of the architecture, not validation of the integrated system.

The next step is staged validation: establish impedance reliability, assess vascular-reference relationships, characterize stimulation artifacts, test fixed-regimen dose–response relationships, and verify the controller before a preregistered comparison of fixed and adaptive treatment. The present architecture is a testable engineering proposal, not a clinically validated system.

## Figures and Tables

**Table 1:** Database yields by query. Counts in the third column are reported as Scopus/PubMed/Web of Science; overlap between queries is retained in the raw totals and removed only in the deduplicated master set.

Query	Main Focus	Scopus/PubMed/WoS	Total Raw
Q1	PEMF and peripheral circulation	57/115/33	205
Q2	PEMF exposure parameters	89/77/23	189
Q3	Bioimpedance and peripheral hemodynamics	96/356/475	927
Q4	Impedance plethysmography/rheovasography	32/120/95	247
Q5	Clinical peripheral applications	62/41/32	135
Q6a	Narrow closed-loop EM therapy	0/0/0	0
Q6b	Broad adaptive/feedback EM stimulation	323/156/218	697
Q7	Endothelial/NO/vascular mechanisms	22/276/281	579
Total	Raw records before cross-query deduplication	681/1141/1157	2979

**Table 2:** Core intervention reports not representable on the shared frequency–flux-density axes of Figure 2 (*n* = 16), grouped by reason. Record identifiers refer to Table S3, which also gives the reference number of each report. NR, not reported; MT, motor threshold; rMT, resting motor threshold.

Reason	Report (Record ID)	Reported Exposure Information	Evidence Role
Incomplete frequency–flux-density pair (*n* = 6)	Lee 2010 [46] (SR-0467)	0.8 T maximum; f NR	Technical/pilot
	Lee 2011 [45] (SR-0461)	≈0.4 T maximum; f NR	Technical/pilot
	Kulchitskaya 2022 [35] (SR-0608)	10 mT; f NR	Direct clinical
	Nawrot 2024 [43] (SR-0143)	0.03–0.10 mT; f NR	Preclinical/veterinary
	Abdelaal 2016 [30] (SR-0355)	10 Hz; B NR	Direct clinical
	Kóródi 2016 [18] (SR-0029)	f and B NR	Clinical support/proxy
Non-equivalent dose metric (*n* = 5)	Tassone 2025 [25] (SR-0002)	27.12 MHz carrier; <3 W forward power	Direct clinical
	Näsi 2011 [47] (SR-1195)	75% rMT; 0.5–2 Hz	Technical/pilot
	Okudera 2015 [39] (SR-1081)	1.2 × rMT; 20 Hz	Clinical support/proxy
	Zhang 2017 [42] (SR-1002)	100–250% MT; 1 Hz	Preclinical/veterinary
	Liu 2019 [38] (SR-0932)	Parameters NR	Clinical support/proxy
Exposure parameters not reported (*n* = 5)	Mohamed 2020 [27] (SR-0018)	NR	Direct clinical
	Biermann 2020 [31] (SR-0272)	NR	Direct clinical
	Kulchitskaya 2017 [33] (SR-1017)	NR	Direct clinical
	Nikolaeva 2010 [34] (SR-1235)	Field-run velocity only	Direct clinical
	Pan 2013 [2] (SR-0036)	NR	Preclinical/veterinary

**Table 3:** Distribution of the 32 core intervention reports across the dimensions that preclude a pooled dose–response relation. Counts were derived from the charting record (Table S3); randomization, sham control, and blinding are summarized separately in Table 4.

Dimension	Category	Reports (n)	Reports (First Author, Year)
Exposure reporting	Reported frequency–flux-density pair	16	Figure 2
	Incomplete pair (one or both values NR)	6	Table 2
	Non-equivalent dose metric (RF power; motor threshold)	5	Table 2
	No exposure parameters reported	5	Table 2
Course length	Single or acute session	10	Sun 2016 [23]; Nawrot 2024 [43]; Mok 2020 [44]; Lee 2010 [46]; Lee 2011 [45]; Liu 2019 [38]; Okudera 2015 [39]; Zhang 2017 [42]; Näsi 2011 [47]; Jo 2018 journal [20]
	Repeated sessions, ≤4 weeks	8	Lu 2017 [40]; Li 2015 [41]; Kwan 2015 [22]; Biermann 2020 [31]; Kulchitskaya 2022 [35]; Kulchitskaya 2023 [32]; Werdan 2024 [37]; Abdelaal 2024 [28]
	Repeated sessions, >4 weeks	10	Alghadier 2026 [24]; Tassone 2025 [25]; Mahmoud 2021 [26]; Ahmad 2021 [29]; Stewart 2020 [4]; Mohamed 2020 [27]; Kóródi 2016 [18]; Abdelaal 2016 [30]; Rikk 2013 [19]; Marković 2016 [36]
	Not reported or unclear	4	Pan 2013 [2]; Kulchitskaya 2017 [33]; Nikolaeva 2010 [34]; Jo 2018 conference [21]
Population or model	Patients with circulatory or metabolic disease	17	Alghadier 2026 [24]; Tassone 2025 [25]; Mahmoud 2021 [26]; Ahmad 2021 [29]; Stewart 2020 [4]; Mohamed 2020 [27]; Sun 2016 [23]; Kwan 2015 [22]; Abdelaal 2024 [28]; Abdelaal 2016 [30]; Kulchitskaya 2023 [32]; Kulchitskaya 2022 [35]; Kulchitskaya 2017 [33]; Nikolaeva 2010 [34]; Werdan 2024 [37]; Liu 2019 [38]; Marković 2016 [36]
	Healthy or ageing volunteers	7	Kóródi 2016 [18]; Rikk 2013 [19]; Biermann 2020 [31]; Okudera 2015 [39]; Näsi 2011 [47]; Lee 2010 [46]; Lee 2011 [45]
	Animal models	5	Lu 2017 [40]; Li 2015 [41]; Pan 2013 [2]; Nawrot 2024 [43]; Zhang 2017 [42]
	In vitro (blood-cell suspension)	3	Mok 2020 [44]; Jo 2018 journal [20]; Jo 2018 conference [21]
Primary circulation endpoint	Microvascular perfusion (LDF, capillaroscopy, MFI, SPP, NIRS)	13	Alghadier 2026 [24]; Tassone 2025 [25]; Kwan 2015 [22]; Pan 2013 [2]; Biermann 2020 [31]; Abdelaal 2016 [30]; Kulchitskaya 2023 [32]; Kulchitskaya 2022 [35]; Kulchitskaya 2017 [33]; Nikolaeva 2010 [34]; Werdan 2024 [37]; Liu 2019 [38]; Näsi 2011 [47]
	Pressure indices or blood pressure (ABI, TBI, MAP)	7	Mahmoud 2021 [26]; Ahmad 2021 [29]; Mohamed 2020 [27]; Abdelaal 2024 [28]; Marković 2016 [36]; Rikk 2013 [19]; Zhang 2017 [42]
	Vessel diameter or flow (ultrasound, FMD)	4	Stewart 2020 [4]; Sun 2016 [23]; Nawrot 2024 [43]; Okudera 2015 [39]
	Temperature proxies	3	Lu 2017 [40]; Li 2015 [41]; Kóródi 2016 [18]
	Pulse-wave or cellular measures (PPG, RBC velocity)	5	Lee 2010 [46]; Lee 2011 [45]; Mok 2020 [44]; Jo 2018 journal [20]; Jo 2018 conference [21]

**Table 6:** Recurring technical characteristics of localized bioimpedance systems relevant to peripheral-circulation monitoring, with interpretive cautions.

Parameter	Reported Examples	Pragmatic Technical Starting Point	Interpretive Caution
Probing frequency	50 kHz [6,7,8]; 100 kHz [5]; multifrequency review [9]	50–100 kHz for pulse recording; multifrequency when justified	Operating frequency does not determine vascular specificity.
Probing current	Approximately 0.8–1 mA in selected systems [5,6,7]	Use the lowest current that meets signal-quality requirements	Compliance depends on the complete device, frequency, isolation, electrodes, and intended use.
Electrode configuration	Tetrapolar localized measurements [5,6,7,8]	Tetrapolar with fixed geometry and documented placement	Contact and segment geometry remain important sources of variation.
Sampling	500 Hz [5]; 838 Hz [8]; 2 kHz [7]	Sufficient bandwidth for fiducial timing and artifact characterization	Higher sampling does not compensate for poor analog filtering or clock alignment.
Recorded quantities	R and Xc [7]; |Z|, Z_0_ and dZ/dt [5,6]	Retain raw or vector components where feasible	Static fluid-related and pulsatile components have different confounders.
Candidate pulse features	Normalized amplitude, pulse arrival time, fiducial timing, DI, area ratios	Prefer a small pre-specified set after repeatability testing	Features are not validated surrogates for capillary flow or eNOS activity.
Electrodes	Standard Ag/AgCl; flexible printed Ag/AgCl [8]	Stable, reproducible contact suitable for the target site	Wearability must not compromise placement repeatability or skin safety.
Anatomical site	Multisegment, knee, calf, foot and toe examples [5,6,7,8]	Measure locally in the treated segment and include a reference site when possible	Distal feasibility does not establish microcirculatory validity.

**Table 7:** Comparative characterization of existing closed-loop and cardiac-synchronized architectures in electromagnetic therapy.

Architecture Element	Cardiac-Synchronized RVG-Guided PEMF [16]	Machnia et al. [78]	Liu et al. [79]	Huang et al. [80]	Mishin et al. [82]	Gurzhin et al. [83]
Exposure	PEMF; cardiac-synchronized timing	PEMF (ONDAMED)	TMS/rTMS	Adaptive EM stimulation	Pulsed magnetic therapy	Chronomagnetotherapy (Multimag complex)
Feedback signal	Rheovasography used for phase selection	Qualitative radial-pulse response	EEG/fMRI/MEG	Physical and position sensors	Four-lead tissue impedance; 30–1000 Hz measuring range; amplitude and phase every 4 s	Photometric pulse/SpO2 monitoring and ultrasound respiratory signals
Adaptation	Manual pre-session test-and-fit	Manual therapist adjustment	Algorithmic analysis and adaptation	Programmable real-time logic	Monitoring only; therapeutic-field adaptation not demonstrated	Bioadaptive regulation discussed; controller performance not demonstrated
Adjusted variables	Cardiac-synchronization phase	Frequency, intensity and duration	Coil position, pattern and intensity	Intensity, frequency and pattern	None	Therapeutic field variables not reported in full text
Time scale	Between sessions/open loop	Between sessions	Potentially real time	Real time	Before, during and after exposure; 4 s stored estimates	On-line monitoring; respiratory phase delay limits synchronization
Target	Peripheral circulation	Peripheral clinical symptom	Central nervous system	Neural experimental models	Pelvic tissue during intravaginal magnetotherapy	Functional (autonomic) patient status
Evidence role	Historical contextual antecedent	Single clinical case	Review/architectural context	Engineering development	Preliminary patient monitoring; sample size and independent perfusion validation not reported	Engineering methods and illustrative signals; no controlled therapeutic outcome evaluation
Relevance	Closest impedance-guided precedent, but not a closed loop	Peripheral-signal individualization	Measurement–analysis–adaptation template	Open sensor/actuator implementation	Early concurrent impedance-monitoring precedent; therapeutic loop not closed	Monitoring and proposed feedback using non-impedance physiological signals

**Table 8:** Observed PEMF exposure and outcome patterns in the core corpus of 32 reports grouped into 30 study/report clusters. Values describe the literature and are not clinical recommendations.

Dimension	Observed in the Core Corpus (32 Reports)	Recurrent Pattern	Interpretation
Frequency/waveform	Low-frequency 10–30 Hz and 15–50 Hz; 27.12 MHz burst RF; 1 Hz high-field technical work	Low-frequency regimens, often near 15 Hz, recur clinically	Frequency alone is not a dose, and no universal optimum is established.
Field/power	Approximately 0.1–5 mT in many clinical low-frequency protocols; 0.02–0.8 T in technical work; RF power reported separately	Low-mT range in clinical studies	B cannot be compared directly across low-frequency, RF, and high-dB/dt regimes.
Session schedule	Acute single exposure to 60 min/session; RF trial 30 min twice daily	24–60 min often reported	No minimum effective session duration is established.
Course length	Single session to 18 weeks	8–12 weeks in several clinical studies	Population, comparator, and co-intervention differences prevent an optimal-course claim.
Populations/models	PAD, diabetic foot/neuropathy, hypertension, healthy volunteers, rodent ischemia, horses, in vitro microchannels	PAD and diabetes dominate clinical relevance	Animal, proxy, and technical evidence should not be pooled with direct clinical perfusion.
Circulation endpoints	LDF, PPG, ABI/TBI, SPP, vessel diameter/velocity, temperature, walking outcomes	Objective instrumental endpoints predominate	Measures address different physiological scales and are not interchangeable.
Outcome direction	Heterogeneous: positive, mixed, and negative results coexist (Section 3.1)	Heterogeneous rather than uniformly positive	Descriptive counts do not estimate efficacy or account for study quality.
Bioimpedance feedback	No core intervention report (of 32) used real-time limb bioimpedance feedback	Technical feasibility only	Simultaneous validation against a reference vascular method is required.

## Data Availability

No new data were created in this study. The search strategies, screening records, charting tables and sensitivity analyses supporting this review are provided in the Supplementary Materials (Tables S1–S6).

## Supplementary Materials

The following supporting information can be downloaded at: https://www.mdpi.com/article/10.3390/bioengineering13101160/s1, Table S1: Complete Boolean search strategies; Table S2: PRISMA-ScR checklist; Table S3: Charting table for 32 core intervention reports (30 study/report clusters); Table S4: Deduplicated screening record; Table S5: Supplementary IEEE Xplore search: independent duplicate screening record; Table S6: Full-text-only sensitivity analysis. References [2,4,10,17,18,19,20,21,22,23,24,25,26,27,28,29,30,31,32,33,34,35,36,37,38,39,40,41,42,43,44,45,46,47,82,83] are cited in the Supplementary Materials.

## Abbreviations

The following abbreviations are used in this manuscript:

PEMF	Pulsed electromagnetic field
LDF	Laser Doppler flowmetry
PPG	Photoplethysmography
IPG	Impedance plethysmography
RVG	Rheovasography
eNOS	Endothelial nitric oxide synthase
NO	Nitric oxide
cGMP	Cyclic guanosine monophosphate
CaM	Calmodulin
TRPV4	Transient receptor potential vanilloid 4
VEGF	Vascular endothelial growth factor
HIF-1α	Hypoxia-inducible factor 1-alpha
DI	Dicrotic index
PWV	Pulse-wave velocity
PAD	Peripheral arterial disease
FMD	Flow-mediated dilation
RCT	Randomized controlled trial
PRISMA-ScR	Preferred Reporting Items for Systematic Reviews and Meta-Analyses extension for Scoping Reviews
ABI	Ankle-brachial index
TBI	Toe-brachial index
SPP	Skin perfusion pressure
